# Object Detection in Computer Vision: A Comprehensive Review of Algorithms, Datasets, and Evaluation Techniques

**DOI:** 10.3390/s26206663

**Published:** 2026-10-21

**Authors:** Md. Faishal Rahaman, Xueyuan Li, Md Nagib Mahfuz Sunny, Muhammad Amjad, Xin Gao, Sakib Hasan

**Affiliations:** 1 School of Mechanical Engineering, Beijing Institute of Technology, Beijing 100081, China; faishalrahaman@bit.edu.cn (M.F.R.); muhammadamjad@bit.edu.cn (M.A.); gaoxin2000@bit.edu.cn (X.G.); 2 Department of Engineering & Technology, Trine University, Angola, IN 46703, USA; nagibmahfuz1996@gmail.com; 3 International Project Execution and Service Center, Qingdao TGOOD Electric Co., Ltd., Qingdao 266104, China; sakib.hasan@qdtgood.com

**Keywords:** computer vision, object detection, deep learning, YOLO, transformer, vision–language model, survey

## Abstract

Object detection has evolved dramatically over three decades, transitioning from handcrafted features to deep learning, transformers, and now large language models. This paper provides a comprehensive systematic review of this evolution, covering traditional methods (SIFT, HOG, Viola-Jones), CNN-based single- and two-stage detectors (R-CNN family, YOLO v1–v12, SSD), transformer-based architectures (DETR, ViT, Swin), and emerging LLM-driven approaches (GPT-4V, Grounding DINO, YOLO-World). We benchmark and compare these methods using key metrics—mAP, IoU, FPS—and analyze over 50 datasets across generic, autonomous driving, medical, remote sensing, and agricultural domains. Our critical synthesis identifies persistent research gaps: lightweight edge deployment, sensor fusion for real-time systems, bias and fairness in AI, and multimodal vision–language integration. By consolidating three decades of progress and outlining future challenges, this survey serves as a valuable resource for researchers and practitioners seeking to develop accurate, efficient, and reliable object detection systems.

## 1. Introduction

Computer vision (CV) is a branch of artificial intelligence that enables machines to extract meaningful information from visual data such as images and videos; image segmentation, image classification, object tracking, and object detection are among its most frequently used tasks [1,2]. Object detection (OD) is a supervised learning task that identifies instances of predefined object classes in images and videos and localizes each instance by drawing a bounding box around it [1,2,3]. Its key difficulty is the semantic gap between human perception and the numerical matrices with which computers represent images, which gives rise to intra-class variation, illumination changes, viewpoint variation, and occlusion [2,4].

**Figure 1:**
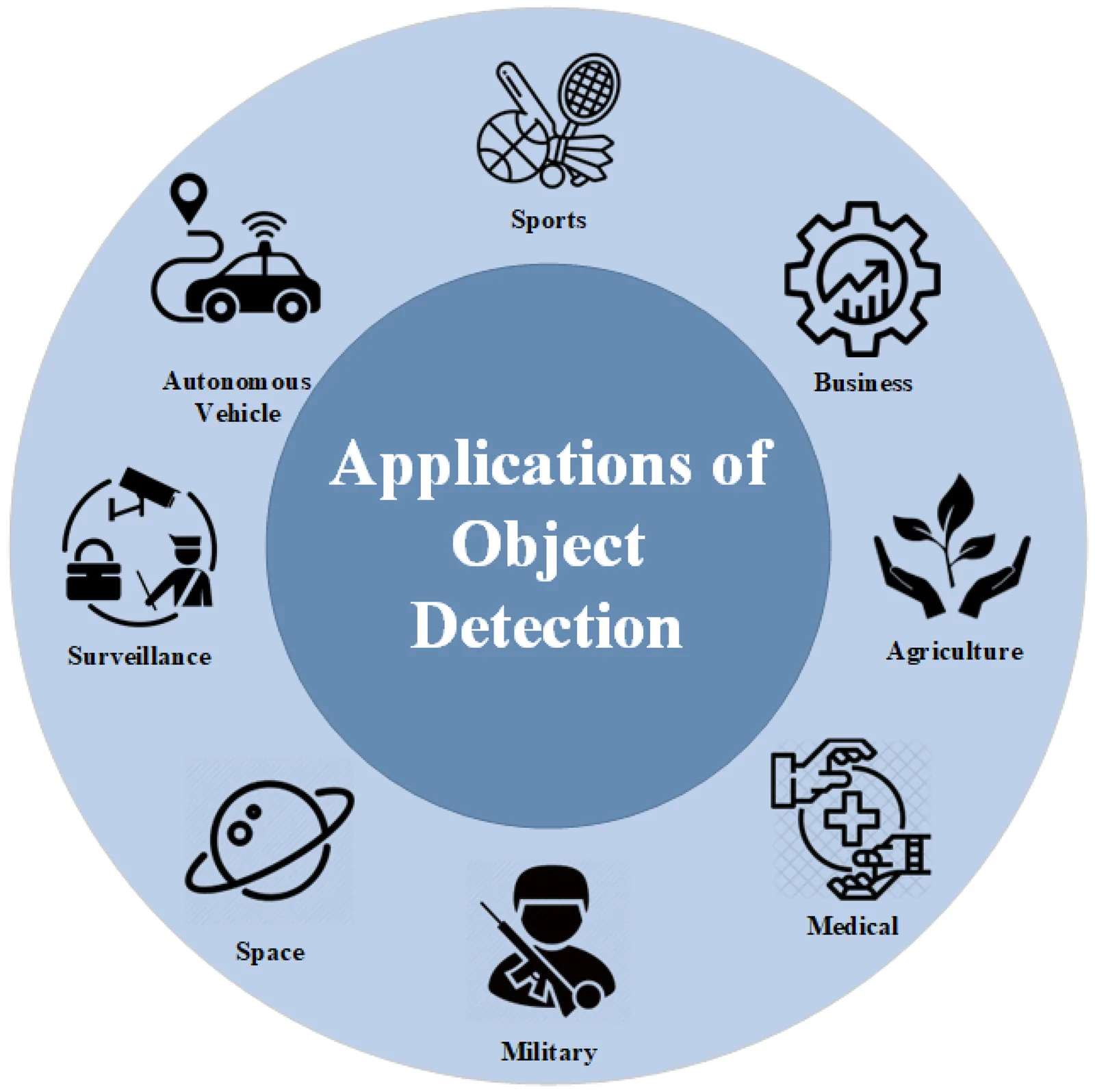
Applications of object detection in different fields and domains.

Despite these challenges, OD has found wide application (Figure 1), and its adoption has grown sharply since the introduction of deep learning (DL). In transportation, it supports traffic analysis and autonomous driving [5,6,7,8,9]; in security and surveillance, it enables video analysis, face recognition, and the localization of suspects and weapons in secured places [1,4,10,11]; and in defense, it helps to detect spy drones, missiles, and intruders near border areas [12,13,14,15]. In medicine, DL-based detection helps identify brain tumors [16,17], internal bleeding [18], lung cancer [19], pneumonia, and COVID-19 [20,21,22] from medical images [23,24,25]. Businesses use OD for industrial quality control, production monitoring, inventory management, fault analysis, and worker safety monitoring [26,27], while e-commerce companies apply it to product delivery, customer support, and compliance [28,29]. In agriculture, it supports intelligent agricultural equipment [30,31], plant disease identification [32], pest classification and prevention [33,34], and crop monitoring [35,36], contributing to a world without hunger and poverty [37]. Other uses include sports, where detectors locate players for computerized game analysis and refereeing [38,39], as well as image generation, event reconstruction, and the measurement of object properties [40].

**Figure 2:**
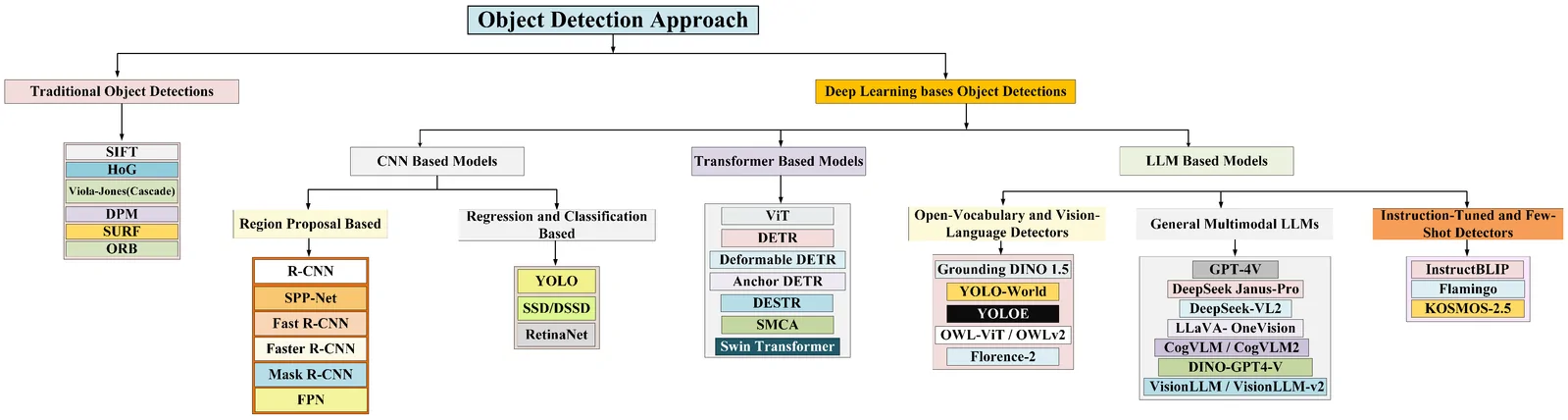
Classification of object detection algorithms.

**Figure 3:**
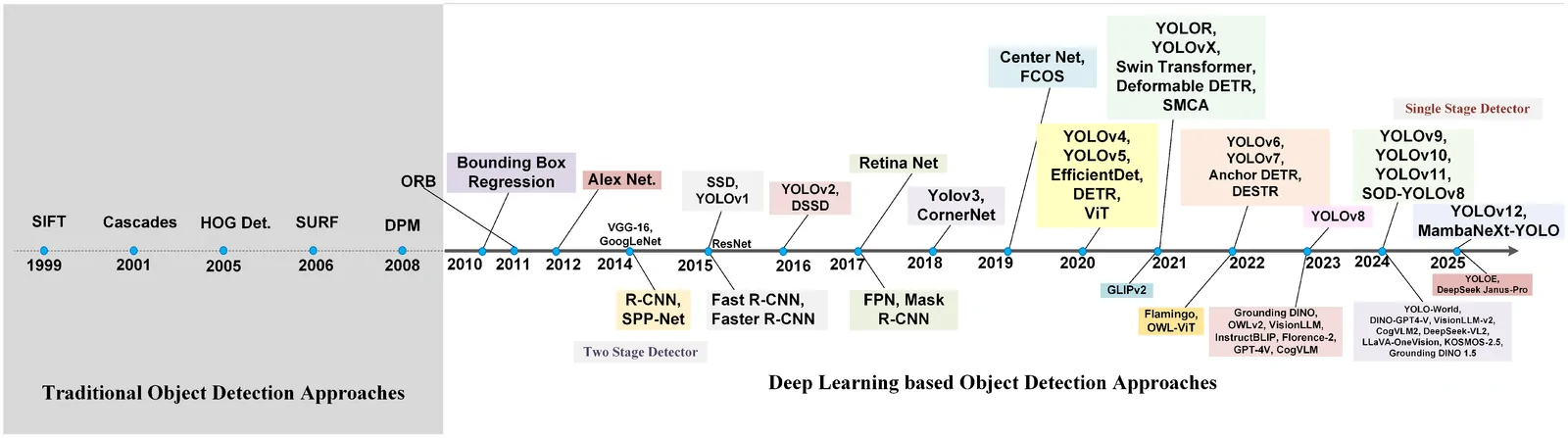
Road map of object detection and the prominent object detection algorithms.

Over the last three decades, OD has developed in two periods (Figure 2 and Figure 3): traditional object detection, and DL-based object detection [41,42,43]. Traditional techniques such as the Viola–Jones detector and Histogram of Oriented Gradients (HOG) rely on manually designed feature extractors and are characterized by slow processing, low accuracy, and poor performance on new or unfamiliar datasets [2,3]. DL-based techniques use convolutional neural networks (CNNs) and transformers; CNN-based detectors are further divided into region proposal-based and regression/classification-based approaches (Figure 2), exemplified by the region-based CNN (R-CNN) and You Only Look Once (YOLO) families [1,2]. Their success has been driven by benchmark datasets such as Pascal Visual Object Classes (Pascal VOC 07/12) [44], OpenImages [45], Microsoft Common Objects in Context (MS COCO) [46], and ImageNet [47] as well as by backbone architectures such as AlexNet [48], VGG-16, GoogLeNet, and ResNet-50 [1,4]. With the rapid emergence of vision–language models [49,50], an up-to-date and concise overview of backbone architectures, object detectors, and benchmark datasets is needed, which this survey aims to provide.

This paper provides a comprehensive review of computer vision-based object detection algorithms and related research trends over the last 30 years, covering traditional algorithms, single- and two-stage detectors, transformer-based and LLM-based detectors, and the evaluation metrics used to assess their accuracy. The key contributions of this review are as follows:

- We propose a structured taxonomy of object detection approaches, covering traditional algorithms, CNN-based architectures, single- and two-stage detectors, transformer-based models, and large language model (LLM)-driven methods.
- We classify benchmark datasets by application domain (e.g., generic, autonomous driving, medical, agricultural) and critically analyze common evaluation metrics such as mAP, IoU, and FPS to in order highlight their implications for accuracy and deployment efficiency.
- We identify emerging challenges, including lightweight edge deployment, multimodal vision–language integration, and fairness in AI for object detection, helping to guide future research and the next wave of object detection systems across different fields.

The rest of the paper is organized as follows: Section 2 outlines the systematic review methodology; Section 3 reviews traditional object detection methods; Section 4 examines CNN-based architectures; Section 5 presents deep learning-based detection methods, covering two-stage, single-stage, transformer-based, and LLM-driven approaches; Section 6 analyzes prominent benchmark datasets; Section 7 discusses evaluation metrics; Section 8 explores real-world applications; Section 9 provides a discussion of challenges and future research directions; and Section 10 concludes the review.

## 2. Review Methodology

**Figure 4:**
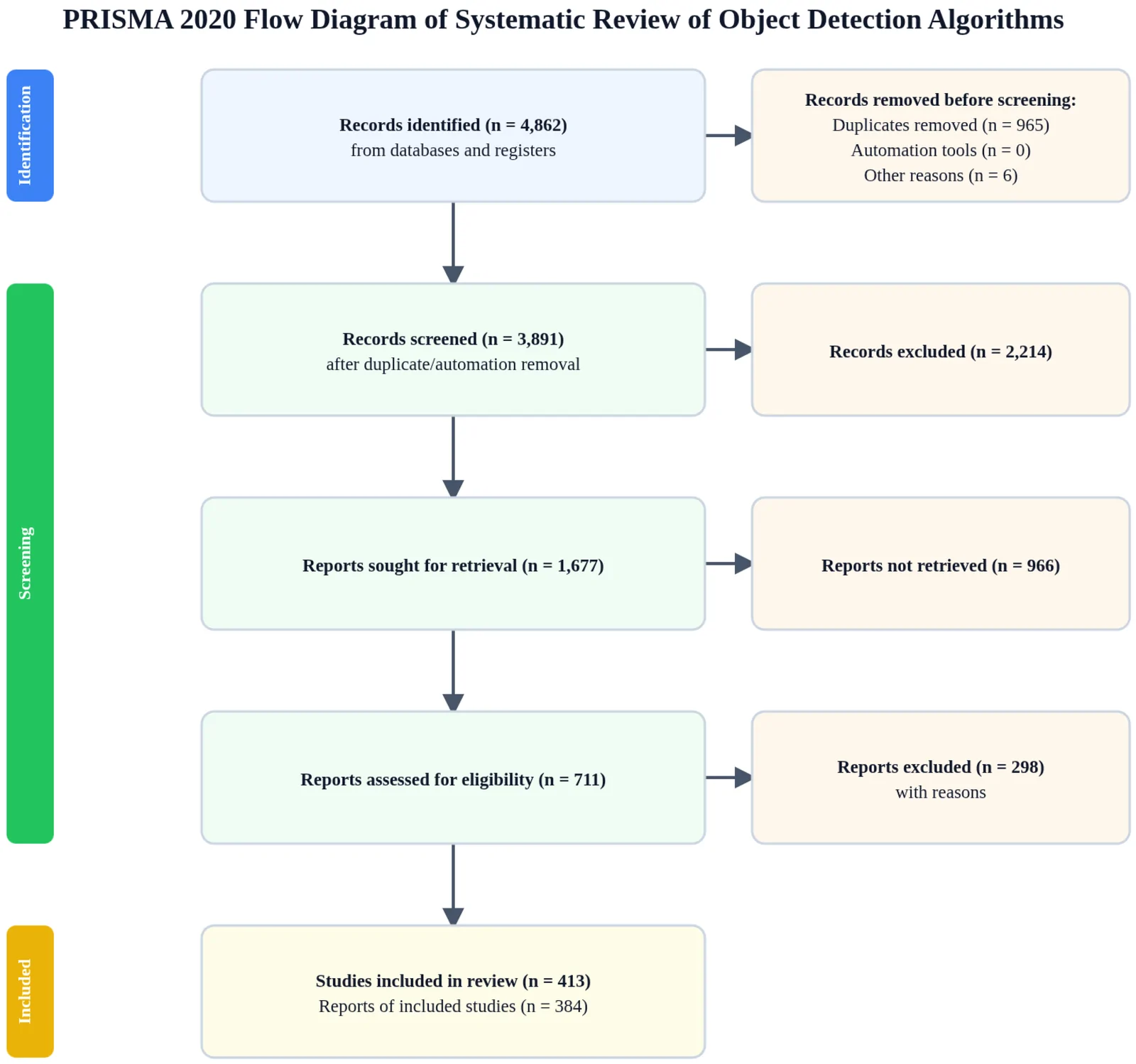
PRISMA 2020 flow diagram of the systematic review.

This systematic review follows the Preferred Reporting Items for Systematic Reviews and Meta-Analyses (PRISMA) 2020 guidelines [51] to ensure methodological rigor, transparency, and reproducibility in synthesizing object detection literature from 1999 to January 2026 (Figure 4). Searches of Google Scholar, IEEE Xplore, and Web of Science yielded 4862 records. After removal of 965 duplicates and exclusion of six records for other reasons, the remaining 3891 records were screened by title and abstract. Of these, 2214 were excluded for irrelevance to object detection, non-English language, or lack of quantitative evaluation. Of the resulting 1677 records sought for retrieval, 966 could not be retrieved, leaving 711 full-text reports assessed for eligibility against the following predefined inclusion criteria: (i) focus on object detection algorithms, (ii) quantitative performance reporting (mAP, IoU, FPS), (iii) novel architectural contributions, and (iv) publication in English. A total of 298 reports were excluded for insufficient methodological quality, incomplete experimental setups, or non-computer vision applications. Of the remaining 413 records, 384 reports met all inclusion criteria and are listed as primary included studies. The additional 29 records, identified through snowballing and forward citation tracking, were consulted for supplementary context but are not listed as primary included studies. Therefore, the final synthesis draws on 384 primary studies supported by 29 supplementary records.

## 3. Traditional Methods of Detection

**Figure 5:**
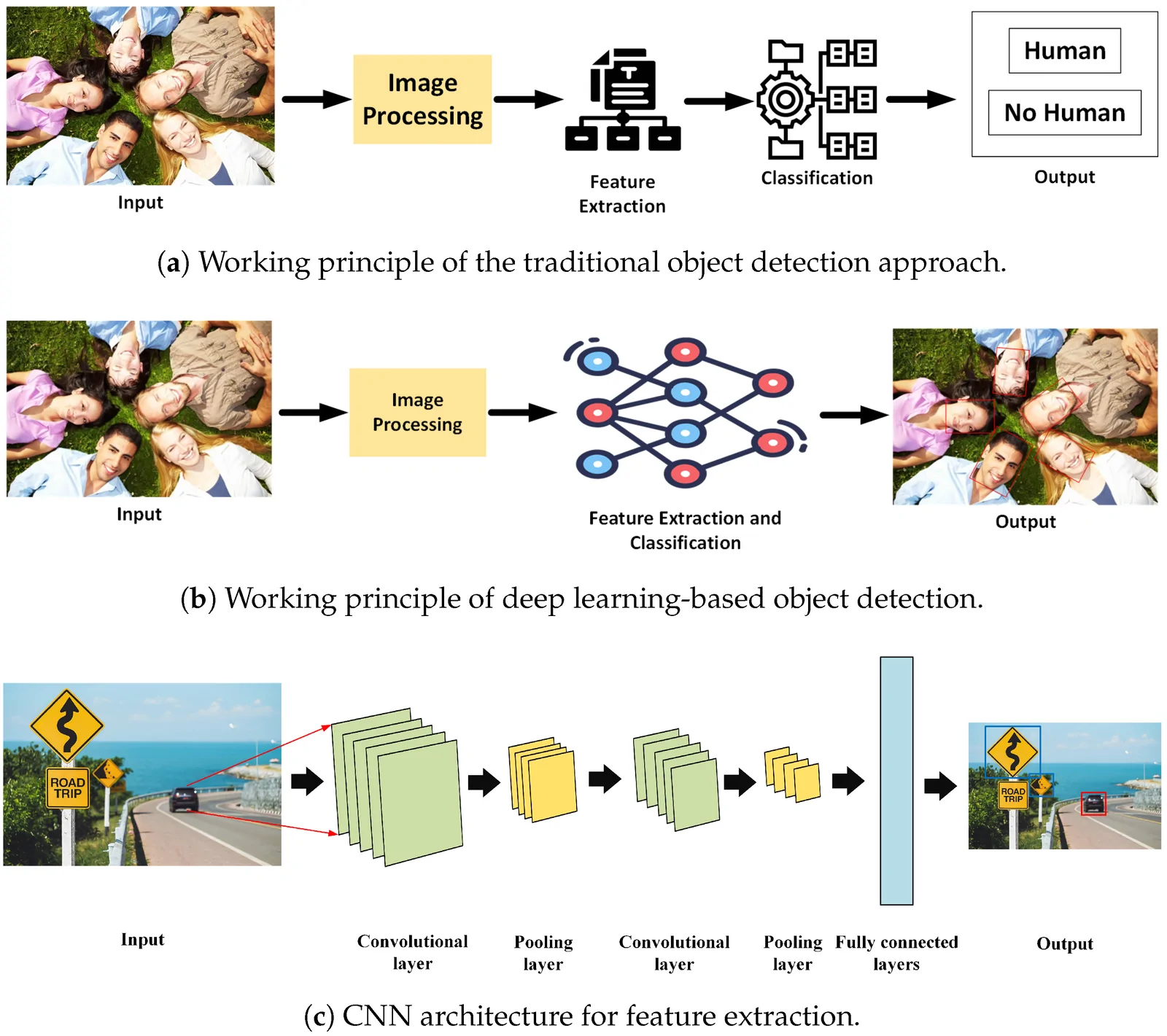
Overview of the object detection pipeline: (**a**) traditional detection workflow, (**b**) deep learning-based detection workflow, and (**c**) CNN architecture for feature extraction and classification.

The traditional period of object detection spans roughly 1999 to 2010 (Figure 3), during which the Scale-Invariant Feature Transform (SIFT), the Histogram of Oriented Gradients (HOG), Speeded-Up Robust Features (SURF), and other hand-crafted approaches were introduced. As shown in Figure 5a, a traditional detector preprocesses the input image (color conversion, resizing, and region selection), extracts image features, and classifies them to determine and localize the object class in the output image. To reduce computational cost, most traditional detectors convert RGB images to grayscale, where feature selection is comparatively easier.

SIFT, proposed by Lowe [52] in 1999, detects and describes local features such as corners and blobs that are invariant to image rotation and scale [53]. It proceeds in four stages: scale-space peak selection to find candidate features, keypoint localization, orientation assignment based on the gradient magnitude and direction around each keypoint to achieve rotation invariance, and keypoint description and matching by nearest-neighbor search [54,55]. Its local features are robust to clutter and occlusion, can be generated in large numbers even for small objects, and can be matched against large databases [56]; for these reasons, SIFT-based methods form a distinct category in image retrieval [57].

**Figure 6:**
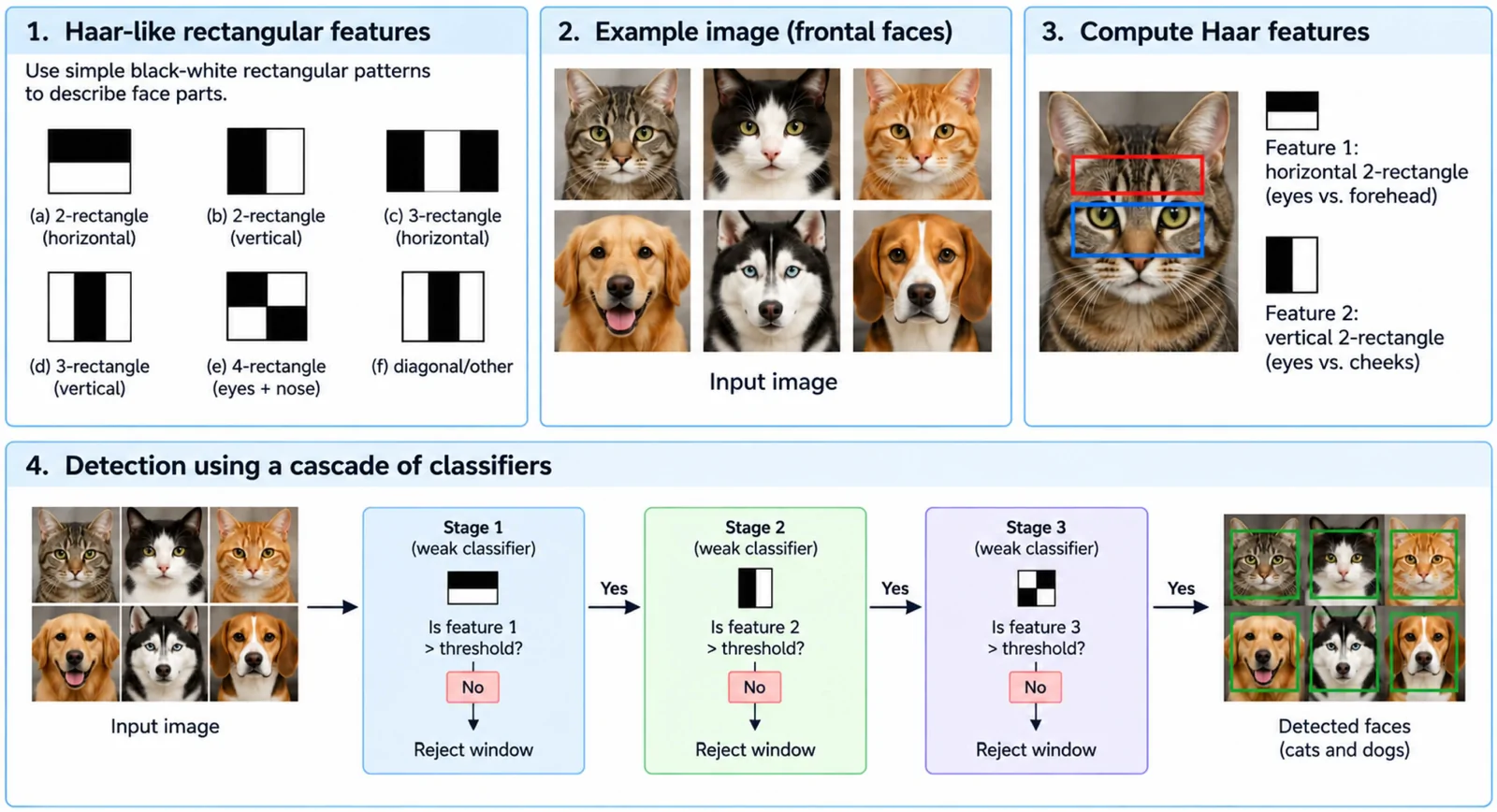
Haar-like rectangular features used in the Viola–Jones face detector for frontal faces of dogs and cats.

The cascade classifier-based Viola–Jones detector, proposed by Paul Viola and Michael Jones in 2001 [58], was the first technique to achieve competitive detection rates in real time. It was mainly used for face detection owing to its high true positive and very low false positive rates [59]; although less accurate than modern CNN- or LLM-based face detectors, it remains an efficient solution for resource-constrained devices. It selects Haar-like features (Figure 6), computes them rapidly from an integral image, classifies facial features using AdaBoost, and combines the resulting classifiers in a cascade that localizes the object. However, its accuracy decreases under varying lighting conditions, and it consumes more processing time than other algorithms [60,61]. HOG, proposed by Navneet Dalal and Bill Triggs in 2005 for human detection [62], divides the image into small cells, constructs a histogram of gradient orientations in each cell, and concatenates the histograms into a descriptor that captures object shape and appearance [53,63,64]. HOG is robust to lighting changes and occlusion. The HOG descriptor can be computed efficiently, making it suitable for real-time applications when combined with classifiers such as Support Vector Machine (SVM) or Random Forests [64], although it performs poorly in textured and cluttered scenes. Beyond pedestrians, HOG can detect other object classes by keeping the detection window fixed and repeatedly rescaling the input image [65]. The Deformable Part Model (DPM), proposed by researchers from the University of Chicago and Toyota Technological Institute, extended the HOG-based approach and won the Visual Object Classes (VOC) Challenge in 2008 and 2009 [66,67]. Following a divide-and-conquer strategy, DPM combines a root filter with multiple part filters, for which the configuration is learned automatically through weakly supervised learning as opposed to being defined manually [68,69]. DPM offers flexibility in representing complex shapes and handling partial occlusion, since each object part is modeled separately. Although outperformed by modern detectors such as Faster R-CNN and YOLO, DPM remains useful for handling deformation and occlusion, and has been improved by combining it with CNN architectures [69] and R-CNN [70] as well as by refining candidate selections via a dense subgraph discovery filter.

SURF, proposed by Herbert Bay and his research group in 2006 [71], is a fast and robust local feature method that is invariant to scale and rotation; its use of box filters enables real-time applications such as object detection and tracking [72,73]. Its pipeline consists of four stages: (1) integral image computation and Fast Hessian interest point detection; (2) keypoint localization and orientation assignment; (3) descriptor construction; and (4) descriptor matching. ORB (Oriented FAST and Rotated BRIEF), proposed by Rublee et al. [74] and developed in the OpenCV lab in 2011, combines the FAST detector with the BRIEF descriptor. It achieves rotation invariance via intensity centroid orientation and uses a Harris measure to prune keypoints; its binary descriptor enables fast Hamming-distance matching, making it faster than SIFT/SURF and well-suited for real-time applications such as SLAM and mobile robotics.

Table 1 summarizes these methods: SIFT and SURF are suitable for object recognition, image retrieval, and 3D reconstruction, the Viola–Jones cascade for real-time frontal face detection and surveillance, HOG for pedestrian and vehicle detection, DPM for handling occlusion and shape variation in cluttered scenes, and ORB for fast binary matching in real-time SLAM and mobile robotics [75].

## 4. Convolutional Neural Networks (CNNs)

After the traditional period, from around 2010, researchers began using convolutional neural network (CNN) architectures for object detection; today, CNNs remain the core component of deep learning-based detectors. As shown in Figure 5b, a deep learning detector first processes the input image to filter distortions, then a CNN extracts features and matches them with object classes in the output image.

A CNN has four crucial layers (Figure 5c). The convolution layer slides filters (kernels) over the input image to produce feature maps that encode edges, boundaries, and other object features. The number of feature maps equals the number of kernels; unlike a regular deep neural network that flattens the image, convolution preserves spatial information [3,4]. The activation layer adds nonlinearity, without which a deep network would reduce to a linear classifier; the widely used Rectified Linear Unit (ReLU) sets negative inputs to zero and passes positive inputs [3,4]. The pooling layer reduces the size of the feature maps, and in turn the computational load, by selecting the maximum, average, or minimum value within local regions. Finally, the fully connected layer, a feedforward network, flattens the feature maps into a one-dimensional vector and performs detection and classification [3,4]. In detection models, the CNN serves as the backbone that extracts feature maps from the input image [1,2,3]. Table 2 compares prominent CNN backbones: AlexNet [48], GoogLeNet [76], VGG-16 [77], and ResNet [78].

AlexNet, proposed by Krizhevsky et al. [48], won the ImageNet Large-Scale Visual Recognition Challenge (ILSVRC) 2012 by a large margin over competing models. It consists of five convolutional and three fully connected layers, and uses ReLU and dropout to increase convergence speed and prevent overfitting [2]. GoogLeNet [76] replaces fully connected designs with a locally sparse architecture to reduce computational cost and parameters; its 22 layers consist of stacked inception modules that apply filters of different sizes in parallel and concatenate their outputs, with auxiliary classifiers in intermediate layers that help to propagate gradients. GoogLeNet achieves high computational efficiency through optimized inception modules, matching the performance of heavier networks on the ImageNet benchmark [2,3]. Simonyan and Zisserman [77] explored the effect of network depth with VGG-16, which stacks small convolution filters that capture a large receptive field with fewer parameters and faster convergence, followed by three fully connected layers and a softmax layer; it outperformed GoogLeNet, the ILSVRC 2014 winner, in single-network performance [2,3]. He et al. [78] introduced skip connections in ResNet; these are element-wise additions between the input and output of a block that add no parameters or computational complexity, and help to counter the degradation of deeper CNNs. Their 101- and 152-layer versions achieved higher accuracy than VGG-16 with lower complexity, and ResNet has inspired many subsequent networks.

## 5. Deep Learning Object Detection Methods

As shown in Figure 2, deep learning-based object detection approaches can be divided into three categories: (1) CNN-based methods, which are further subdivided into region proposal-based (two-stage) detectors and regression/classification-based (single-stage) detectors; (2) transformer-based detectors; and (3) LLM-based detectors [2,79,80]. Two-stage detectors (TSDs) first generate candidate regions and then classify and localize objects from features extracted from these regions, whereas single-stage detectors complete the task in a single attempt by dense sampling, using predefined boxes or key points of different scales and aspect ratios instead of regions of interest [1,2,81]. TSDs are generally more accurate, but are slower, while single-stage detectors are time-efficient and well suited for real-time applications. Transformer-based detectors replace or complement CNNs with the self-attention mechanism of transformers, originally developed for natural language processing, to capture the global context and relationships across the entire image [82,83].

### 5.1. Two Stage Detectors

**Figure 7:**
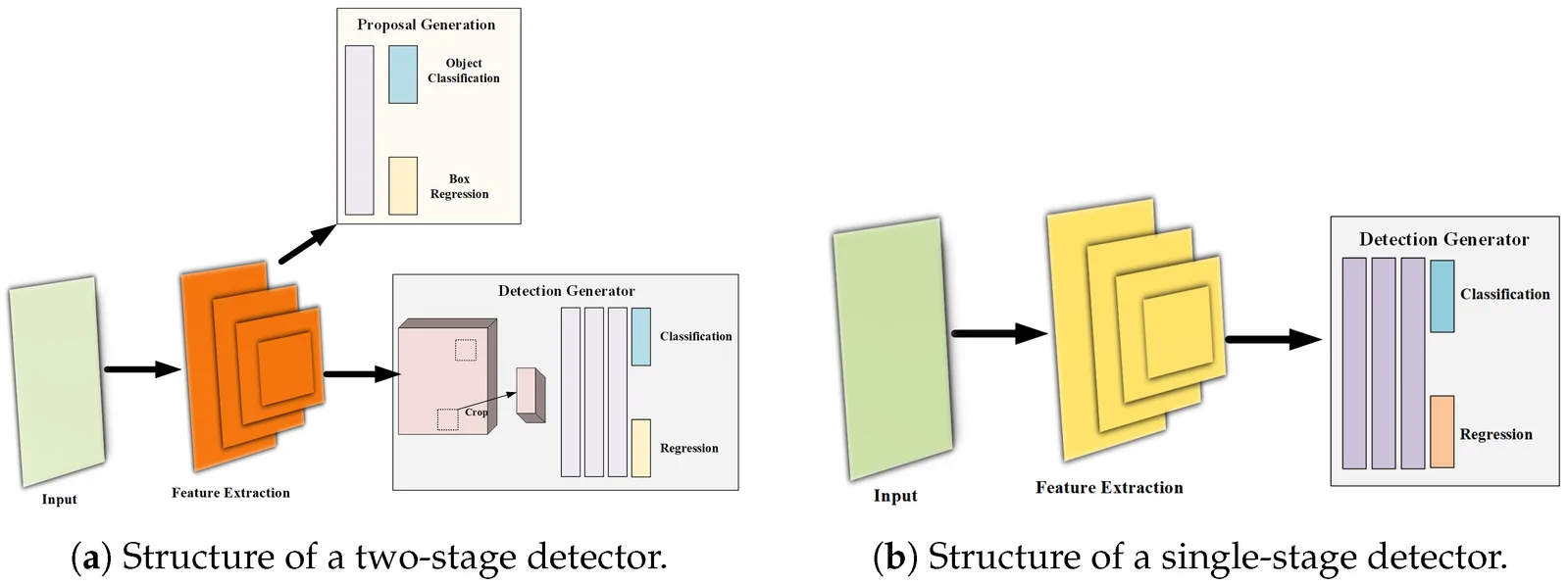
Architectural comparison of two-stage and single-stage object detection pipelines: (**a**) two-stage detector with proposal and detection generator; (**b**) single-stage detector with dense sampling in a single forward pass.

TSDs decouple detection into sequential steps (Figure 7a): the first stage proposes a sparse set of potential object locations, or regions of interest (RoIs), and the second performs fine-grained classification and localization refinement on features extracted from these regions. This focused design yields higher accuracy at the cost of processing speed [1,2,81]. Because they rely on region proposals, TSDs are also called region proposal-based detectors; representative examples include R-CNN, Fast R-CNN, Faster R-CNN, Mask R-CNN, and Spatial Pyramid Pooling Network (SPP-Net), several of which have also been combined with other algorithms [2].

**Figure 8:**
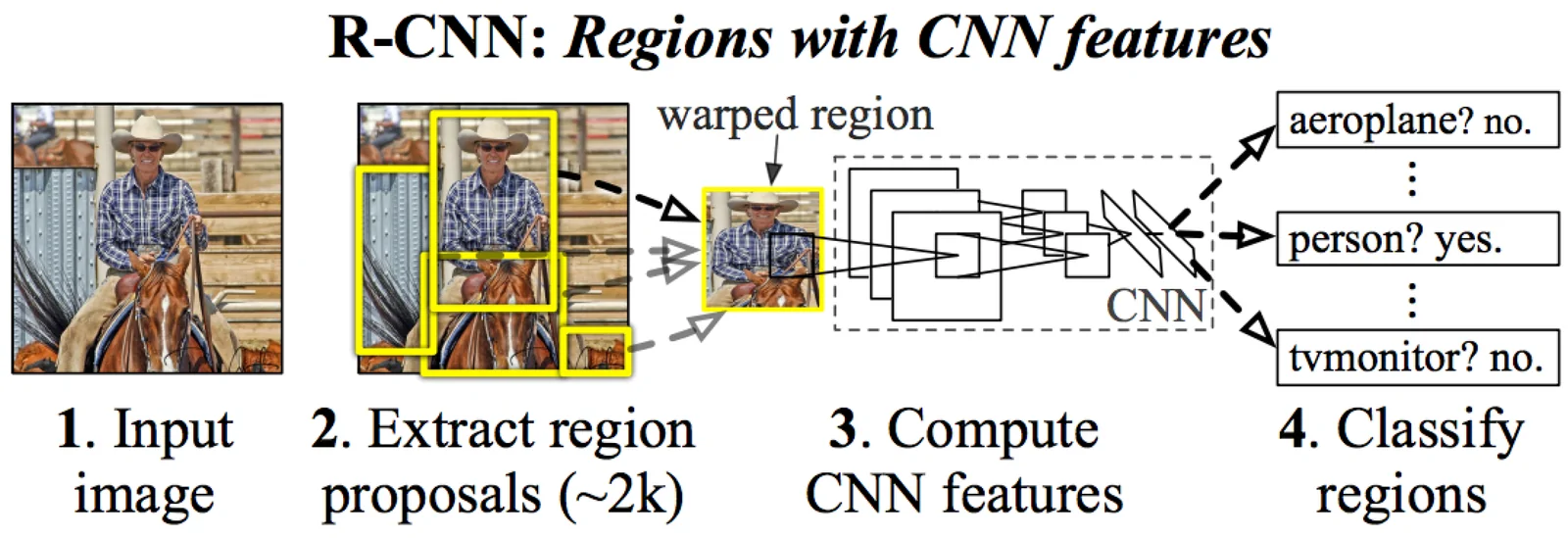
Architecture of R-CNN object detection [86].

R-CNN [84], proposed in 2014, was the first region-based TSD; using the AlexNet [48] architecture, it demonstrated how CNNs can substantially increase detection performance. The R-CNN pipeline first extracts region proposals using selective search, then the detector classifies each proposal using a CNN. Specifically, selective search [85,86] generates about 2000 RoIs, i.e., areas with a high likelihood of containing an object, from the mean-subtracted input image (Figure 8); each RoI is warped to 227 × 227 pixels and passed through five convolutional and two fully connected layers that compute a 4096-dimensional feature vector [1,2,81]. Class-specific Support Vector Machines (SVMs) score each region, non-maximum suppression (NMS) based on the intersection over union (IoU) removes duplicates, and a bounding box regressor refines the box center, width, and height [2,81]. R-CNN is characterized by slow performance (approximately 47 s per image on a single GPU with a VGG-16 backbone [87]) and is both time- and memory-intensive, a limitation that becomes more severe as the dataset scale increases [84,88].

Fast R-CNN [87], proposed in 2015, replaces the multi-stage training of R-CNN (pretraining, fine-tuning, SVM classification, and bounding box regression) with a single multi-task loss applied to each labeled RoI. It extracts features once from the complete image and uses an RoI pooling layer to obtain fixed-size features for each proposal, which fully connected layers then classify and regress [89]. Fast R-CNN has been found to accelerate R-CNN by a factor of 146 [1], improve mAP on PASCAL VOC 2007 from 66.0% to 66.9%, and reduce training time from 84 to 9.5 h [44]. Because selective search remains the bottleneck of Fast R-CNN [2], Faster R-CNN [90], proposed three months later, introduced the Region Proposal Network (RPN), a fully convolutional network that shares full-image convolutional features with the detection network and efficiently predicts proposals at multiple scales and aspect ratios using multi-scale anchors, without requiring image or feature pyramids [1,2]. With the same VGG [77] backbone, Faster R-CNN achieved 69.9% mAP on the PASCAL VOC 2007 test set (versus 66.9% for Fast R-CNN) and reduced the processing time from 1830 ms to 198 ms per image, i.e., from 0.5 to 5 FPS [1].

Mask R-CNN [91] extends Faster R-CNN by adding a mask head alongside the classification and bounding box heads. The mask head predicts pixel-level instance segmentation for each RoI at low additional cost. In addition, Mask R-CNN replaces RoI pooling with RoI alignment to prevent pixel-level misalignment caused by spatial quantization [1,53]. Its advantages include ease of training and good adaptability to tasks such as keypoint detection and human pose estimation, outperforming other single-model architectures [2]. However, Mask R-CNN typically operates at approximately 5 FPS, falling short of the 30 FPS benchmark generally associated with real-time performance, and requires detailed pixel-level annotations for training [2,92].

SPP-Net [93] removes the fixed input size constraint of CNNs, which otherwise necessitates resizing that distorts images or loses information. Inspired by spatial pyramid matching [76,94], the convolutional layers first process an input of arbitrary size into feature maps that retain its aspect ratio [95]; the SPP layer then divides each map into sub-regions at several grid resolutions, max-pools each sub-region, and concatenates the outputs into a fixed-length vector for the fully connected layers [96]. This improves efficiency by avoiding repeated convolution over multi-scale inputs and preserves spatial hierarchies, yielding more robust features for multi-scale recognition [97]. In 2017, Facebook AI Research (FAIR) proposed the feature pyramid network (FPN), which constructs multi-scale hierarchical feature maps through a top-down pathway with lateral connections [98]. A bottom-up pathway produces progressively lower-resolution features while the top-down pathway upsamples semantically strong deep features and merges them with the spatially precise features of earlier layers, producing an output that is rich in both detail and context [98,99,100]. Unlike traditional image pyramid schemes that repeatedly resize the input, FPN builds a rich multi-scale representation from a single input image at negligible additional cost. Because it is independent of the backbone, FPN has been integrated into many detectors [2,101,102,103].

### 5.2. Single Stage Detector

Single-stage detectors perform detection in a single forward pass (Figure 7b): instead of an explicit RoI proposal step, they predict object locations and classes simultaneously over a dense grid of anchor boxes or key points with varying scales and aspect ratios. Their prioritizing of speed and simplicity makes them ideal for real-time applications such as video analysis, robotics, and edge devices [104]. Although their accuracy has historically trailed that of TSDs, architectures such as YOLOv7 [105] enable comparably high accuracy in many scenarios. Table 3 contrasts the two families; the most prominent single-stage detectors are YOLO, SSD, and RetinaNet.

**Figure 9:**
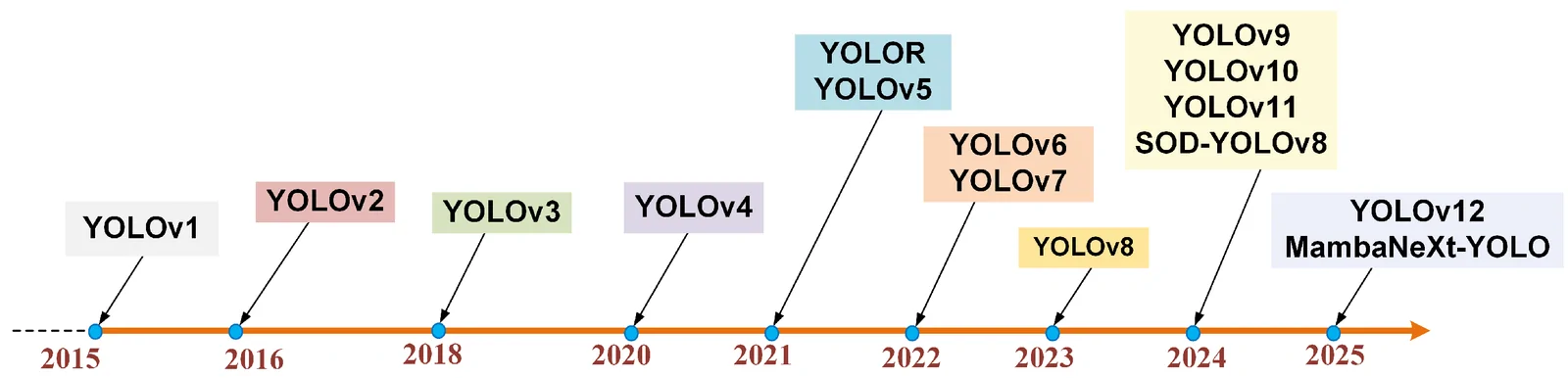
Road map of the YOLO object detector family.

**Figure 10:**
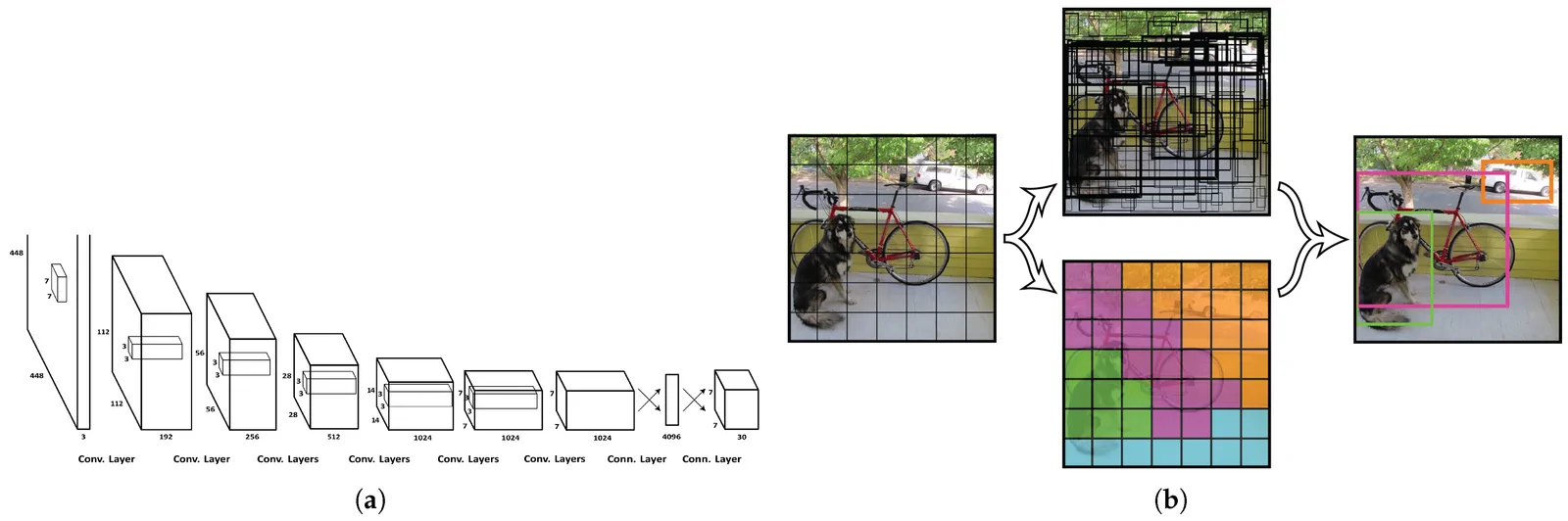
Overview of the YOLO object detector [106]: (**a**) network architecture and (**b**) basic working process.

The You Only Look Once (YOLO) family of detectors has evolved from YOLOv1 in 2015 [106], which introduced real-time detection as a single regression problem, to YOLOv12 [107], with continuous improvements in speed, accuracy, and deployment (Figure 9). Whereas region-based approaches such as R-CNN [1,2,81], Fast R-CNN [44], and Faster R-CNN generate region proposals before classification, YOLO frames detection as a single regression problem [108]: the input image, resized to 448 × 448, is divided into an S×S grid, and each cell directly predicts bounding box coordinates, confidence scores, and class probabilities [109]. The original backbone of 24 convolutional layers with four max-pooling layers followed by two fully connected layers (Figure 10a,b) ran at over 45 FPS, far surpassing earlier detectors [109], but struggled with multiple small objects, irregular bounding boxes, and objects near grid boundaries [110].

YOLOv2 (YOLO9000, 2016) [111,112] replaced the fully connected layers with anchor boxes, improving the handling of multiple objects and irregular aspect ratios [106], and adopted the Darknet-19 backbone with batch normalization, high-resolution classification, multi-scale training, and fine-grained features; joint training on ImageNet and COCO enabled the detection of over 9000 categories [112]. YOLOv3 (2018) [113] adopted Darknet-53, a deeper backbone with residual connections, independent logistic classifiers for multi-label classification, and predictions at three scales, improving the detection of small, medium, and large objects at a higher computational cost. YOLOv4 (2020) [114] combined a CSPDarknet-53 backbone with spatial pyramid pooling (SPP), the Path Aggregation Network (PANet), Mosaic augmentation, DropBlock regularization, IoU loss, cross mini-batch normalization, and Mish activation, achieving state-of-the-art results without expensive hardware. In parallel, YOLOR (2021) [115] combined explicit and implicit knowledge to capture contextual dependencies, and YOLOX [116] introduced a fully anchor-free design with decoupled heads and SimOTA label assignment, reducing dependence on hand-crafted anchors [117]. YOLOv5 (2020), released by Ultralytics in PyTorch [118], became one of the most widely adopted versions owing to its modular design, community support, multiple model sizes (s, m, l, x), efficient augmentation, and suitability for lightweight deployment in applications ranging from robotics to surveillance [119].

YOLOv6 (2022) [120] targeted industrial and edge use with hardware-friendly backbones, an efficient decoupled head, and advanced label assignment. YOLOv7 (2022) [105] introduced the Extended Efficient Layer Aggregation Network (E-ELAN) and a trainable bag of freebies, outperforming contemporary transformer- and CNN-based detectors at real-time speeds [117]. YOLOv8 (2023) [121] adopted an anchor-free head, simplifying training and inference while improving accuracy for small and occluded objects [122,123] to support classification, detection, and segmentation with flexible model scaling [46,124]. SOD-YOLOv8 [125] is an enhanced YOLOv8-based model designed for small object detection in aerial imagery and traffic scenes. It integrates an attentional scale sequence fusion (ASF) mechanism in the neck for multi-scale feature fusion, adds a small object detection layer (P2) for higher-resolution feature maps, and employs Soft-NMS to refine confidence scores, achieving a 36.1% increase in mAP50:95 on the VisDrone2019-DET dataset compared to the YOLOv8 baseline.

YOLOv9 (2024) [126] introduced Programmable Gradient Information (PGI) and Generalized ELAN (GELAN), improving gradient flow and parameter efficiency, although extreme occlusion, densely packed small objects, and imbalanced datasets remained difficult. YOLOv10 (2024) [127] eliminated NMS through consistent dual assignment, reducing the postprocessing latency. YOLOv11 (2024) [128] added the C3k2 block and the C2PSA module, combining cross-stage partial connections with self-attention to capture global context. Finally, YOLOv12 (2025) [107,129] fully embraces attention, using area attention, Residual ELAN (R-ELAN), an anchor-free head, Mosaic, MixUp and copy–paste augmentation, and FlashAttention to achieve notable gains in small object detection and occlusion robustness [130,131], although optimal deployment requires specialized hardware. Beyond YOLOv12, MambaNeXt-YOLO [132] represents a hybrid direction that integrates CNNs with the linear-complexity Mamba state space model through a dedicated MambaNeXt Block, enabling efficient capture of both local features and long-range dependencies. It further introduces a multi-branch asymmetric fusion pyramid network (MAFPN) for improved multi-scale detection, achieving 66.6% mAP at 31.9 FPS on PASCAL VOC while remaining deployable on edge devices such as NVIDIA Jetson Xavier NX and Orin NX.

Table 4 summarizes the YOLO family. Taken together, YOLO has progressed from grid-based regression to anchor-based frameworks, then to anchor-free detection, and finally to attention-driven architectures [133], with each version addressing specific limitations of its predecessor. Along the way, YOLOR’s hybrid implicit–explicit representation was eventually surpassed in speed and generalization by newer versions [119], whereas YOLOX’s decoupled anchor-free head and SimOTA assignment laid the foundation for its successors. The series shows a clear trend toward unified, efficient, and multitask-capable models: YOLOv7 and YOLOv8 added task alignment learning and multitask outputs such as instance segmentation and pose estimation, while YOLOv12 adopts a generalist approach that performs detection, segmentation, and pose estimation with a unified decoupled head and an NMS-free design [109,134]. These models balance high accuracy with real-time processing, making them central to autonomous driving, robotics, surveillance, and edge AI [107,135]. Future developments of the YOLO family are expected to include the following: (1) researchers will continue to refine YOLO architectures using cutting-edge deep learning techniques, data augmentation, and improved training approaches to enhance performance, resilience, and efficiency in a continuous cycle of innovation; (2) evaluation will use more challenging benchmarks than COCO 2017, mirroring the earlier shift from VOC 2007; (3) more frequent releases will address a wider range of applications, from home appliances to autonomous cars; (4) extension beyond detection and segmentation will cover video object tracking and 3D keypoint estimation; and (5) deployment across hardware platforms from IoT devices to high-performance computing clusters will make YOLO accessible to more users and industries.

**Figure 11:**
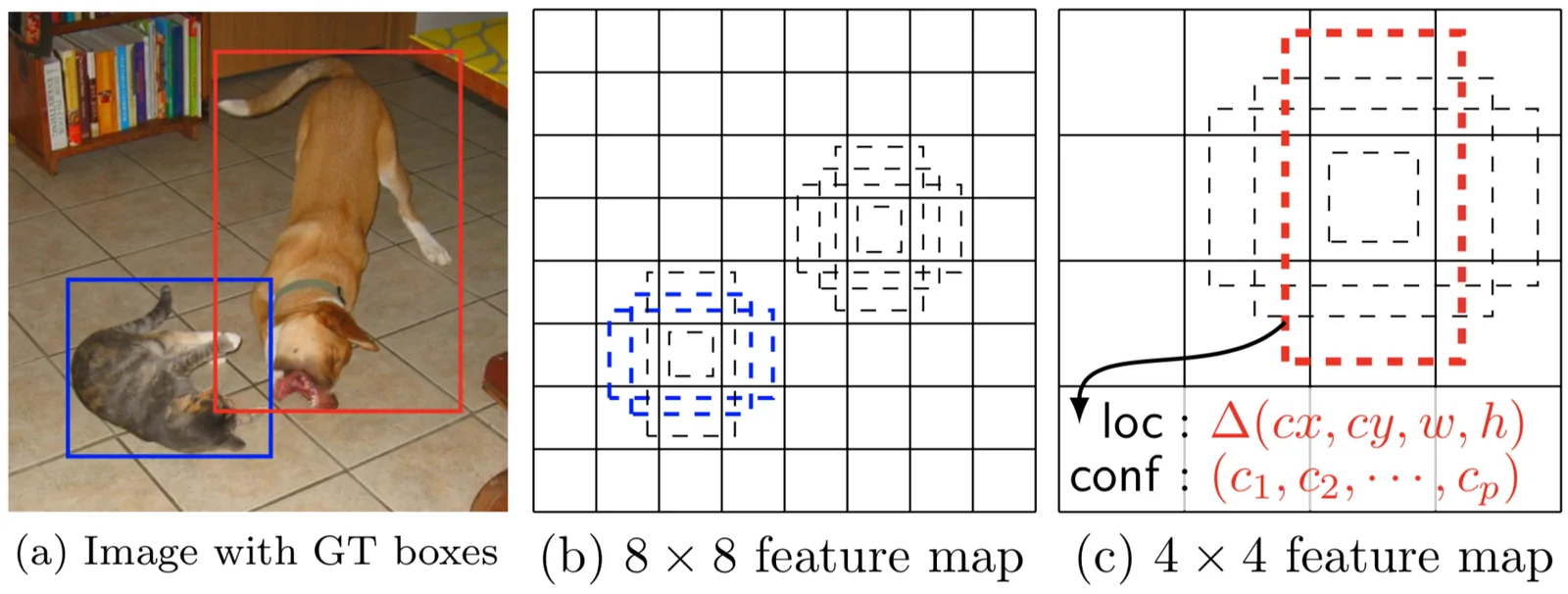
Process of the SSD object detector [136].

The first single-shot detector (SSD) was published by Wei Liu et al. [136] in 2015, shortly after YOLO, and was later refined [137]; in 2017, the deconvolutional single-shot detector (DSSD) introduced further modification [138]. Figure 11 shows the working process of the SSD object detector; rather than dividing the image into a grid, as YOLO does, it predicts offsets of predefined anchor boxes at every location of multiple feature maps. The anchor boxes tile each feature map convolutionally, and because feature maps at different levels have different receptive fields, each level is responsible for objects of a particular scale [136].

RetinaNet [139], introduced by Facebook AI Research in 2017, addresses the extreme foreground–background class imbalance of dense single-stage detectors [140]. In two-stage frameworks such as Faster R-CNN, the RPN reduces candidate locations to a sparse set (e.g., 1–2k proposals), while sampling heuristics such as fixed foreground–background ratios or online hard example mining (OHEM) balance training [50,139]; on the other hand, single-stage detectors evaluate over 100k candidate locations per image. RetinaNet’s focal loss adds a tunable modulating factor to the cross-entropy loss that decays as confidence in the correct class increases, thereby downweighting easy examples and focusing the training on hard misclassified ones without explicit sampling [141,142,143]. The network comprises a backbone (e.g., ResNet) that extracts multi-scale feature maps, a classification subnet for dense category prediction, and a box regression subnet, both of simple convolutional design [50].

### 5.3. Transformer-Based Object Detection

**Figure 12:**
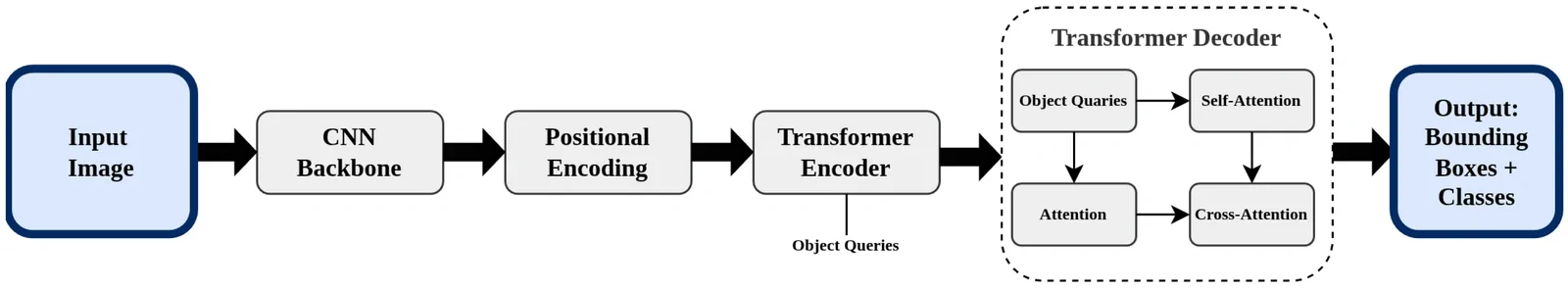
Simplified architectural block diagram of a transformer-based object detector (e.g., DETR), highlighting the sequential flow from feature extraction to direct set prediction.

Rather than relying solely on CNNs, transformer-based detectors leverage the self-attention mechanism of the transformer architecture, originally developed for natural language processing (NLP), to capture global context and long-range relationships between objects across the entire image, which contributes to their robustness [82,83]. As shown in Figure 12, DETR-style detectors are built on direct set prediction: a CNN backbone extracts a reduced-resolution semantically rich feature map, which is then positionally encoded, flattened, and fed to a transformer encoder. The transformer’s multi-head self-attention creates dependencies across the entire scene [144]. The decoder refines a fixed set of learned object queries through self- and cross-attention; feedforward prediction heads then output class labels and bounding boxes directly, eliminating heuristics such as NMS and anchor design [145].

**Figure 13:**
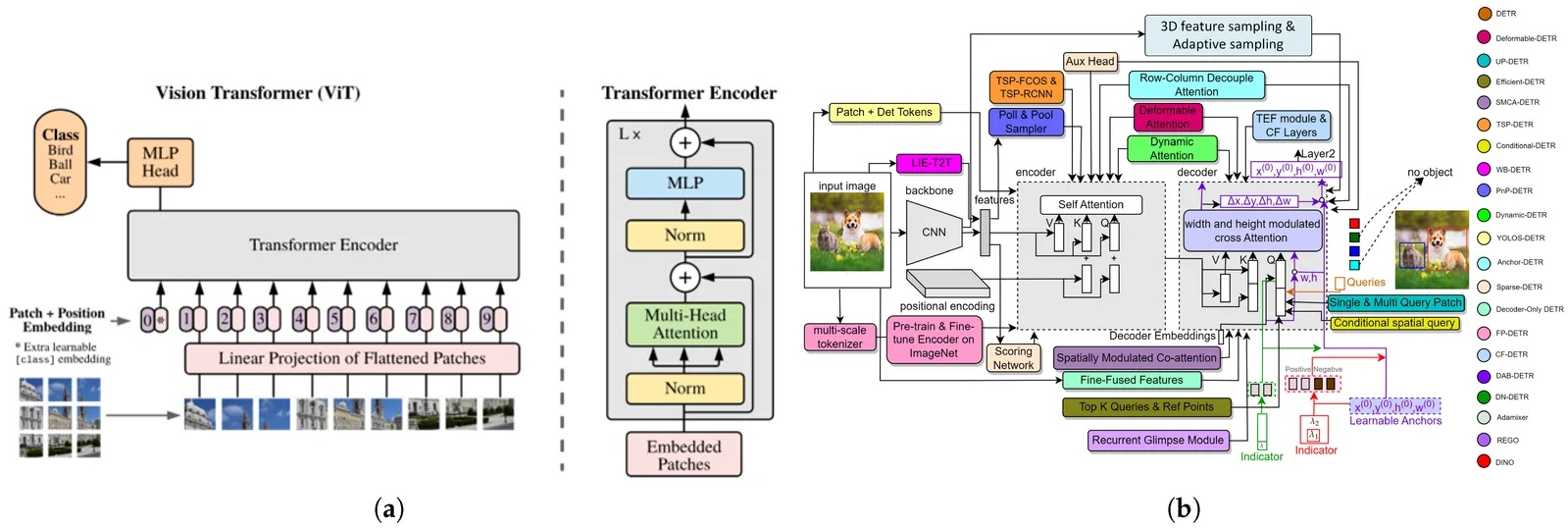
Overview of transformer based detectors: (**a**) basic architecture of Vision Transformer (ViT), as mentioned by the Google research team [146]; (**b**) Detection Transformer (DETR) and modifications proposed by recent methods to improve its performance and training convergence [158].

Vision Transformer (ViT) [146] adapts the standard transformer to images by dividing the input into fixed-size patches (e.g., 16 × 16 pixels), linearly projecting each patch into an embedding, and processing the resulting token sequence with a transformer encoder (Figure 13a). Unlike CNNs, which rely on local receptive fields and hierarchical aggregation, ViT captures global dependencies across the entire image [147,148,149], which is valuable in dense scenes with multiple objects [150]. Lacking the inductive biases of CNNs, such as locality and translation invariance, it requires very large datasets and extensive computational resources. Although designed for classification, ViT is widely used as a backbone combined with detection heads or region-based architectures [151,152], and has laid the foundation for more advanced transformer-based detectors [153,154,155,156,157].

DEtection TRansformer (DETR), introduced by Carion et al. in 2020 [159], is a fully end-to-end transformer framework that eliminates hand-designed components such as anchor generation, region proposal networks, and NMS (Figure 13b). A CNN (typically ResNet) extracts visual features, the transformer encoder processes them globally with self-attention, and the decoder predicts a fixed number of objects from learned queries, each producing a bounding box and a class. A set-based global loss using Hungarian bipartite matching assigns predictions uniquely to ground-truth objects, thereby avoiding duplicate detections. DETR converges slowly, requires many training epochs and large datasets, and struggles with small or overlapping objects [160]; nevertheless, its formulation of detection as direct set prediction has inspired many improved models. Swin Transformer [161] combines transformers’ global modeling with CNNs’ hierarchical feature processing. Self-attention is computed within non-overlapping local windows that are cyclically shifted between layers, reducing the quadratic cost of standard attention while preserving long-range dependencies, and image patches are progressively merged to produce multi-scale feature maps (e.g., 1/4, 1/8, and 1/16 resolution), emulating FPNs [98]. This hierarchical processing enables Swin Transformer to achieve superior performance in dense prediction tasks. As a backbone for Mask R-CNN [162] or Cascade Mask R-CNN [163], Swin achieves state-of-the-art results on COCO [46]; Swin-Large reached 58.7 box AP on COCO test-dev, surpassing ResNeXt-101-FPN by 4.5 AP [161], and variants from Swin-Tiny to Swin-Large cover resource-constrained to high-accuracy applications. Spatially modulated co-attention (SMCA) [164] addresses DETR’s slow convergence and diffuse attention by modulating the co-attention maps in the decoder with a Gaussian prior centered on each query’s reference point, ensuring that each query focuses on regions likely to contain an object [151]. This significantly accelerates convergence and improves detection of small objects and objects in crowded scenes while retaining DETR’s end-to-end set prediction [158]. Anchor DETR [165] initializes object queries from predefined anchors, which provide positional priors that the decoder refines. By combining the strengths of anchor-based and transformer-based detection [82], this improves convergence speed and detection of objects with widely varying scales while preserving DETR’s end-to-end design [166,167]. Deformable DETR [168] introduces a deformable attention module that allows each query to attend to a sparse set of sampling points around a reference location rather than the entire feature map [169,170]. This sparsity reduces computational complexity, focuses attention on relevant regions of small or partially occluded objects, and supports multi-scale feature fusion across backbone levels [82,167], yielding significantly faster training and higher accuracy while preserving DETR’s set-based prediction with bipartite matching. We note that DESTR [171] is a distinct architecture built on split cross-attention and mini-detector query initialization, and should not be confused with Deformable DETR. Table 5 compares these transformer-based detectors with the YOLO family.

### 5.4. LLM Based Models

**Figure 14:**
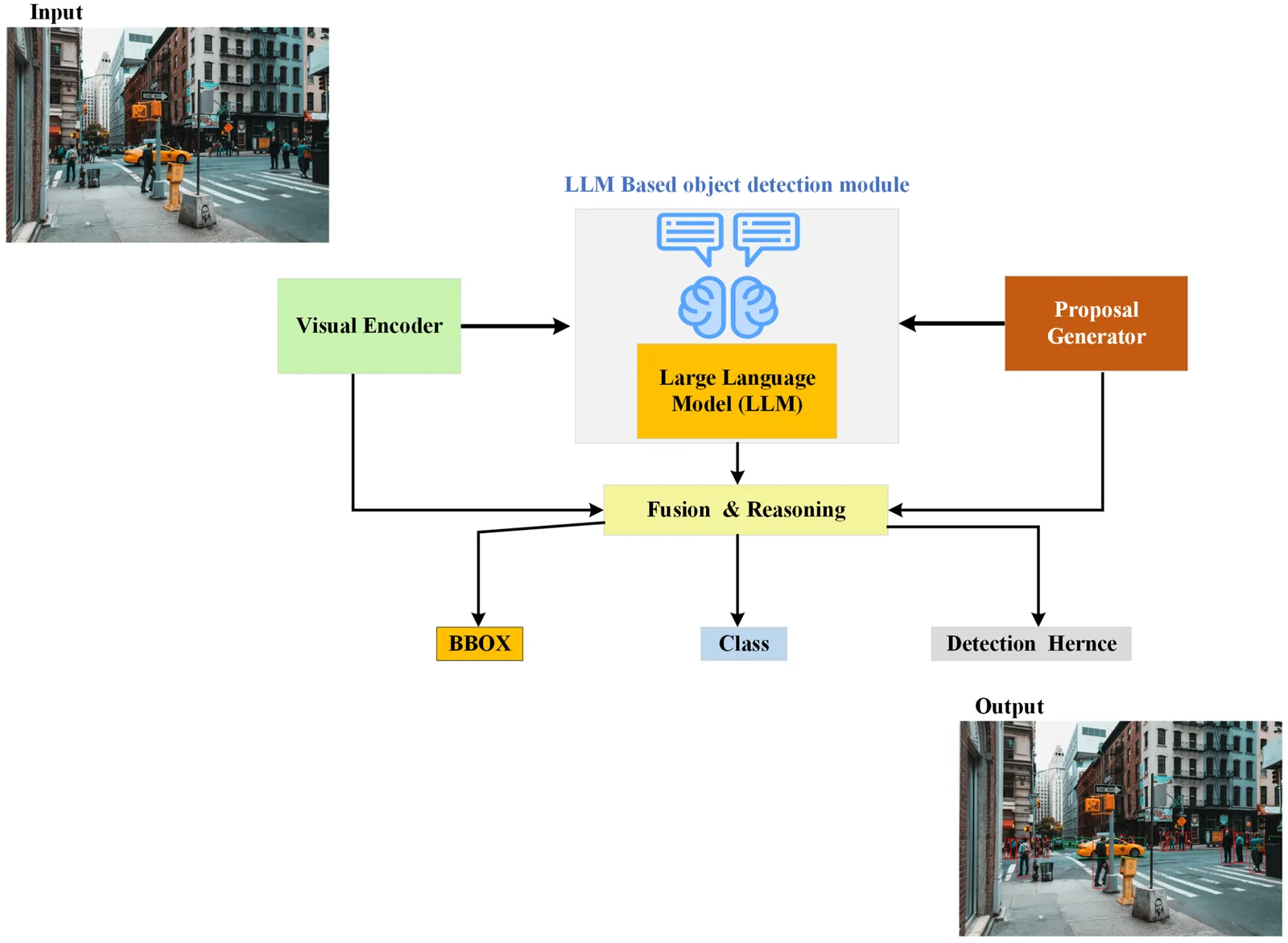
Overview of the LLM-based object detection approaches.

Large vision–language models (LVLMs) have redefined object detection by leveraging the reasoning, compositional understanding, and zero-shot generalization of LLMs [172]. Unlike traditional detectors trained on closed-vocabulary datasets, they align text and images through large-scale multimodal pretraining, enabling open-vocabulary recognition, spatial grounding, and contextual reasoning; they often have billions of parameters, and range from encoder-only to encoder–decoder architectures. As shown in Figure 14, the input image is analyzed by the LLM together with a visual encoder and a contextual proposal generator, which help the model to detect the object class, draw the bounding box (BBOX), and enhance detection accuracy [172]. LLMs can interpret natural language queries to localize objects, reason about object relationships, and describe scenes [173], with applications in autonomous driving, medical imaging, remote sensing, and human–AI interaction. Because these models differ substantially in architecture and task formulation, we group them into three subcategories: open-vocabulary and vision–language detectors, general multimodal LLMs, and instruction-tuned and few-shot models. Table 5 compares transformer-based detectors, Table 6 compares open-vocabulary and foundation models, and Table 7 summarizes the LLM-based detectors discussed in this section.

#### 5.4.1. Open-Vocabulary and Vision–Language Detectors

Grounding DINO 1.5 [174], developed by IDEA Research, is an open-set detector with two variants. The Pro model scales up the vision backbone and is trained on 20 million grounding-annotated images, achieving 54.3 AP on COCO and 55.7 AP on LVIS-minival zero-shot transfer [175]. The Edge model reduces feature scales for efficiency and, optimized with TensorRT, runs at 75.2 FPS with 36.2 AP zero-shot on LVIS-minival, balancing accuracy and deployment efficiency from cloud pipelines to edge devices [176,177].

YOLO-World [178] extends YOLO from closed-set to open-vocabulary detection [106,119]. Its Reparameterizable Vision–Language Path Aggregation Network (RepVL-PAN) fuses image features with text embeddings while preserving YOLO’s real-time efficiency [110], and a region–text contrastive loss aligns the two modalities. When pretrained on large-scale detection, grounding, and image–text data, it achieves 35.4 AP at 52 FPS on LVIS on a single V100 GPU; after fine-tuning, it also performs open-vocabulary instance segmentation and visual grounding [178].

YOLOE [179] unifies detection and segmentation in a single framework that supports text prompts, visual prompts, and a prompt-free mode. It introduces Reparameterizable Region–Text Alignment (RepRTA), which refines text embeddings with a lightweight auxiliary network at no inference cost; a Semantic-Activated Visual Prompt Encoder (SAVPE) with decoupled semantic and activation branches; and Lazy Region–Prompt Contrast (LRPC), which uses a built-in large vocabulary to identify objects without costly external language models. On LVIS [180], YOLOE-v8-S outperforms YOLO-Worldv2-S [178] by 3.5 AP with 1.4× faster inference and three times lower training cost; on COCO, YOLOE-v8-L surpasses the closed-set YOLOv8-L by 0.6 AP^*b*^ and 0.4 AP^*m*^ with nearly 4× less training time [177].

OWL-ViT [181] builds an open-vocabulary detector on a standard Vision Transformer [146] contrastively pretrained on large image–text datasets, as in CLIP. With minimal architectural changes, it supports zero-shot text-conditioned queries (e.g., “a red car”) and one-shot image-conditioned queries, performing strongly on LVIS [180]; however, limited box-level annotations constrain its performance on rare categories. OWLv2 [182] addresses this with the OWL-ST self-training recipe, in which an existing detector generates pseudo-box annotations on web-scale image–text data (10M to over 1B pairs), combined with pseudolabel filtering and efficient training. OWLv2 with the L/14 architecture improves zero-shot detection on LVIS rare categories from 31.2% to 44.6% AP [182]. Its limitations include the high cost of training on billions of images, pseudolabel noise, and weaker fine-grained localization than task-specific detectors.

Florence-2 [183] is a vision foundation model with a unified prompt-based sequence-to-sequence architecture in which text prompts specify tasks such as detection, captioning, visual grounding, and segmentation [184]. It is pretrained on FLD-5B, comprising 126 million images and 5.4 billion annotations produced by an iterative pipeline of automated annotation and model refinement, and performs region proposal, phrase grounding, and detection without task-specific architectures [185], achieving state-of-the-art zero-shot and fine-tuning performance across multiple benchmarks [176,177,186]. Table 6 compares YOLOv8 [121], a strong closed-set real-time baseline; YOLO-World [178], which adds open-vocabulary capability; Florence-2, a large-scale prompt-based foundation model; and YOLOE, which integrates text, visual, and prompt-free mechanisms in a single real-time framework [179].

**Table 6:** Comparison of YOLOE, YOLO-World, YOLOv8, and Florence-2 object detectors.

Model	Architecture	Prompt Mechanism	Key Innovations	Datasets/Training	Performance Highlights	Advantages
YOLOv8 [121]	Convolutional YOLO framework with CSP-based backbone and PAN-FPN head	Closed-set (predefined categories only)	Anchor-free design, decoupled head, mosaic augmentation	COCO and related closed-set datasets	High accuracy and speed in closed-set detection	Real-time, efficient, lightweight, widely used baseline
YOLO-World [178]	YOLO + vision-language integration via RepVL-PAN	Text prompts (open vocabulary)	RepVL-PAN (vision–language fusion), region–text contrastive loss	Pretrained with detection, grounding, and large image-text datasets	35.4 AP @ 52 FPS on LVIS (V100)	Open-vocabulary, strong zero-shot, efficient real-time
Florence-2 [183]	Transformer-based unified vision foundation model	Text prompts (task instructions)	Sequence-to-sequence prompt-based multi-task learning	FLD-5B (126M images, 5.4B annotations)	Strong zero-shot detection, grounding, segmentation, captioning	Foundation-scale, versatile across CV and V&L tasks
YOLOE [179]	Unified detection + segmentation YOLO framework	Text, visual, and prompt-free	RepRTA (text alignment), SAVPE (visual prompts), LRPC (prompt-free vocab)	LVIS, COCO, large vocabulary datasets	+3.5 AP vs YOLO-Worldv2-S on LVIS, 1.4× faster, 3 × less training cost	Multi-prompt support, real-time “see anything,” efficient training

#### 5.4.2. General Multimodal Large Language Models

GPT-4V, introduced by OpenAI, is the first multimodal member of the GPT family, extending the generative pretrained transformer to images and text. Unlike YOLO [106,119], which uses specialized detection heads, or DETR [159], which formulates detection as direct set prediction, GPT-4V is a foundation model pretrained on large-scale multimodal data and fine-tuned with reinforcement learning from human feedback (RLHF), enabling zero- and few-shot detection from natural-language prompts. Multimodal alignment layers connect visual embeddings with textual representations [187], allowing it to localize objects and reason about their relationships and scene context in ways that YOLO, Faster R-CNN [90], and DETR cannot. However, it suffers from hallucinations, high computational cost, and less precise localization than specialized detectors [177].

DeepSeek Janus-Pro [188] improves on Janus with optimized training, larger-scale data, and larger models for both multimodal understanding and visual generation. It decouples visual encoding within a unified autoregressive transformer: a SigLIP encoder [189] extracts semantic features for understanding, and a VQ-based tokenizer [190] discretizes images for generation, with adaptors mapping both into the LLM input space. This enables object localization together with reasoning about object relationships, although the 384 × 384 input resolution limits fine-grained visual detail [177].

DeepSeek-VL2 [176] is a mixture-of-experts (MoE) vision–language model that combines a SigLIP-SO400M-384 encoder, a vision–language adaptor, and a DeepSeekMoE language model with multi-head latent attention, which compresses the key–value cache for efficient inference. A dynamic tiling strategy splits high-resolution images of varying aspect ratios into 384 × 384 tiles plus a global thumbnail, supporting dense tasks such as OCR, infographic understanding, and fine-grained localization. Whereas GPT-4V often struggles with fine-grained spatial grounding [187], while KOSMOS-2.5 relies on fixed input resolutions and LLaVA-NeXT lacks the efficiency of MoE designs, DeepSeek-VL2 activates only 1.0B, 2.8B, or 4.5B parameters while delivering competitive or state-of-the-art performance. Its limitations include a relatively short multimodal context window and reduced performance on blurry or unseen objects.

LLaVA-OneVision [191], developed from the LLaVA-NeXT series, is an open large multimodal model that unifies single-image, multi-image, and video scenarios by connecting a vision encoder to a decoder-only LLM trained on large curated image–text data. It supports instruction-driven detection and multi-turn reasoning with spatial grounding; unlike InstructBLIP [192], which relies on Q-Formers, or Flamingo [193], which focuses on few-shot adaptation, it achieves strong cross-scenario transfer without multiple task-specific detectors [194], performing competitively against larger closed-source systems [195].

CogVLM [196] replaces the shallow vision–language alignment of models such as BLIP-2 with deep fusion, inserting a trainable visual expert module into the attention and feedforward layers of the LLM without degrading its language performance. CogVLM-17B achieved state-of-the-art results on seventeen multimodal benchmarks, including image captioning, visual question answering, and visual grounding (RefCOCO, RefCOCOg). CogVLM2 [197] (2024) supports inputs up to 1344 × 1344 pixels and includes CogVLM2-Video for temporal grounding. Remaining limitations include hallucination, the need for better supervised fine-tuning (SFT) [198] and RLHF [199], and high computational overhead.

DINO-GPT4-V [187] builds on DINO (DETR with Improved deNoising anchOr boxes) [174], an end-to-end transformer detector that, like DETR [159], comprises a backbone, a transformer encoder–decoder, and prediction heads; DINO-GPT4-V adds contrastive denoising training with hard negatives, mixed query selection for anchor initialization, and a look-forward-twice scheme for gradient flow [200]. DINO alone achieved 49.4 AP (12 epochs) and 51.3 AP (24 epochs) on COCO with a ResNet-50 backbone, surpassing DN-DETR by +6.0 AP and +2.7 AP, respectively, and reached 63.3 AP on COCO test-dev with a Swin-L backbone pretrained on Objects365 [201]. Building on this foundation, DINO-GPT4-V combines DINO’s detection capability with GPT-4V’s multimodal reasoning for enhanced contextual understanding [202].

Although LLMs exhibit strong zero-shot generalization and user-adaptive capabilities [203,204], vision foundation models remain largely confined to predefined tasks [172]. VisionLLM [205] addresses this gap by treating images as a foreign language: a unified language instruction interface defines vision tasks, a language-guided image tokenizer encodes visual content, and an LLM-based open-ended decoder generates task-specific predictions [206]. It attains over 60% mAP on COCO, which is comparable to task-specific detectors but with far greater flexibility. VisionLLM-v2 [207] integrates perception, understanding, and generation using a super-link mechanism between the multimodal LLM and task-specific decoders to mitigate multi-task conflicts, and extends to object localization, pose estimation, and image generation [172].

#### 5.4.3. Instruction-Tuned and Few-Shot Detectors

InstructBLIP [192] extends BLIP-2 with an instruction-aware Query Transformer (Q-Former) that extracts visual features tailored to the given task instruction. Trained on 26 public datasets converted to instruction-tuning format, it outperforms BLIP-2 [208], Flamingo [193], and MiniGPT-4 [209] in zero-shot generalization and can adapt to user-defined detection tasks (e.g., “detect all vehicles and pedestrians in the scene”) without retraining, making it a promising foundation for instruction-driven detection in autonomous driving, robotics, and surveillance [176,177].

Flamingo [193] bridges pretrained vision-only and language-only models to process arbitrarily interleaved images, videos, and text. It adapts to new tasks through in-context few-shot learning without gradient-based retraining, which suits detection in low-data regimes and generalization to unseen object categories [210]. Compared to contrastive models such as CLIP, it adapts better to open-ended and structured tasks [211], although hallucinations, sensitivity to in-context examples, and computational cost remain challenges.

**Table 7:** Comparison of LLM-based object detection models, showing their key contributions, efficiency, limitations, and technical features.

Model	Key Contributions	Efficiency	Limitations	Technical Features
GPT-4V [187]	Open-vocabulary detection, bounding box descriptions with reasoning	Scalable, real-time	Coarse localization, needs external grounding	Parameter count not officially disclosed (industry estimates ∼1T–1.8T), CLIP-ViT-L encoder, decoder-only
DeepSeek JanusPro [188]	High-resolution small-object localization, pretrained from scratch	Efficient at 7B	Training data undisclosed, unclear generalization	SigLIP-Large-Patch16-384, decoder-only
DeepSeek-VL2 [176]	Multi-task detection + captioning via MoE	Scalable with 74 experts	High computational cost	4.5B × 74 MoE, SigLIP + SAM-B, decoder-only
Kosmos-2.5 [212]	Zero-shot detection with spatial tokens	Lightweight (1.3B)	Limited fine-grained detection	ViT-L encoder, unigram tokenizer, enc-dec
InstructBLIP [192]	Instruction-tuned for versatile VQA + detection	Strong generalization	Not specialized for detection	13B, ViT + Flan-T5/Vicuna, enc-dec
LLaVA-OneVision [191]	Bounding box outputs in VQA	Mid-scale (7B)	Limited dataset scale	CLIP-ViT-L + LLaMA-2, decoder
Grounding DINO1.5 [174]	LLM-integrated zero-shot detection (47.7 mAP)	Compact (110M)	Focused on detection, less reasoning	Swin-B + BERT, encoder
Florence-2 [183]	Unified captioning + detection	Moderate (5B)	Requires FLD-900M scale	ViT-g + T5, enc-dec
YOLO-World [178]	Real-time open-vocab detection (52 FPS, V100)	Highly efficient (42M)	Limited by YOLO backbone	YOLO-CSP + CLIP, encoder
YOLOE [179]	Prompt-free zero-shot detection	Real-time, efficient	Weaker reasoning than LVLMs	1.3B, RepRTA + SAVPE, enc-dec
Flamingo [193]	Advanced open-vocab reasoning	High-capacity (80B)	Computationally heavy	ALIGN/M3W, Chinchilla backbone, decoder
CogVLM [196]	Contextual multimodal reasoning	Strong at 18B	Resource intensive	CLIP-ViT-L/14 + Vicuna, enc-dec
OWL-ViT v2 [181]	Efficient open-vocab detection (47.0 mAP)	Lightweight (630M)	Limited LLM reasoning	ViT-B/16 + BPE, encoder
DINO-GPT4-V [187]	Two-stage refinement with GPT-4	Mid-scale (1.2B)	Requires GPT-4 integration	DINOv2 + GPT-4, hybrid
VisionLLM [172]	Unified prompt-based detection	Moderate (13B)	Not specialized for small objects	ViT-L + LLaMA, decoder

KOSMOS-2.5 [212] from Microsoft is a multimodal literate model for text-intensive images. Unlike conventional detectors such as YOLO [109] or DETR, it uses a decoder-only autoregressive transformer pretrained on 357.4 million document pages to generate spatially-aware text blocks with bounding box coordinates and structured Markdown output, thereby enabling zero-shot text detection and grounding for document parsing, infographic understanding, and scene–text localization. Its fine-tuned variant, KOSMOS-2.5-CHAT, matches much larger models (1.3B vs. 7B parameters) on nine text-rich VQA benchmarks [177]. Compared with GPT-4V, which struggles with precise layout comprehension, and DeepSeek-VL2, which emphasizes high-resolution tiling [176], KOSMOS-2.5 specializes in literate multimodal intelligence, although multi-page documents and long-context reasoning remain challenging.

Table 8 compares the four families of deep learning detectors in terms of their performance trade-offs. Two-stage detectors, typified by the Faster R-CNN family, prioritize accuracy through serial processing: the first stage generates sparse region proposals, while the second stage performs bounding box regression and category classification for each proposal. This makes them the most accurate, especially for small and occluded objects, though at the cost of multi-step latency. Single-stage detectors such as the YOLO family perform detection in a single forward pass with dense sampling, making the pipeline simpler and computationally cheaper; although historically less accurate, current versions have reached near-parity and are the standard when time is critical [158]. Transformer-based detectors use self-attention to model global context and perform end-to-end set prediction, often without heuristics such as NMS. In addition to being highly accurate and robust, recent variants can also reach real-time speeds, making them competitive in general-purpose detection where global context is paramount [145,158]. Finally, LLM-based detectors combine vision with a language model [172]; despite having the greatest architectural complexity and latency, they enable open-vocabulary and zero-shot detection by using linguistic cues to infer object presence and position, including for classes never observed during training. The choice among these paradigms is ultimately a trade-off among latency, precision, and application scope [172].

## 6. Datasets

Datasets play a pivotal role in object detection: large-scale annotated image collections provide the data for training deep learning models and serve as standardized benchmarks for fair comparison of competing algorithms [213]. Curated datasets such as Pascal VOC, MS COCO, and ImageNet have enabled increasingly accurate and generalizable detectors, while domain-specific datasets (e.g., BDD100K for autonomous driving or CrowdHuman for surveillance) support specialized applications. Persistent challenges include limited diversity, label noise, class imbalance, dataset bias, and high annotation cost, which often lead to poor generalization in real-world unconstrained environments [108]; emerging remedies include semi- and self-supervised learning, synthetic data generated with generative models and simulators, and active learning that prioritizes the most informative samples for annotation [83]. Based on their use, we group benchmark datasets into generic, autonomous driving (including pedestrian detection), remote sensing, face detection, medical imaging, and agricultural datasets [83].

### 6.1. Generic Object Detection Datasets

Generic datasets such as PASCAL VOC, MS COCO, and Open Images provide the annotated imagery required to train, validate, and benchmark detectors. They established standardized evaluation metrics such as mean average precision (mAP) [2,83]. Their evolution, marked by increasing volume, diversity, and annotation complexity, has directly driven performance breakthroughs, particularly with the advent of deep learning. Models trained on these datasets underpin autonomous navigation, robotic vision, image retrieval, video surveillance, and content moderation; Table 9 provides a comparison.

Caltech-101 [214], introduced in 2003 by the California Institute of Technology, is a benchmark for image classification and categorization containing 9146 images in 101 object categories (e.g., faces, ants, pianos, and watches) plus a background category, with object outline annotations; images were retrieved from Google Images and manually filtered. Caltech-256 [215] (2006) extended it to 30,607 images in 256 categories and increased the minimum number of images per category from 31 to 80 while avoiding artifacts from rotated images, and also added a larger clutter category for background rejection; it was benchmarked with methods such as spatial pyramid matching [42].

The PASCAL VOC datasets [44], developed from 2005 to 2012, cover 20 categories (e.g., birds, bottles, persons, bicycles, and dogs) grouped into vehicles, animals, household objects, and people, with approximately 11,000 images, over 27,000 annotated object instances and almost 7000 detailed segmentations. In VOC2007, the “person” class has nearly 20 times more training instances than the smallest class, “sheep” [1,2].

MS-COCO [46] is one of the most challenging detection benchmarks. It contains 80 object categories (complemented by 91 stuff categories), captured in their natural context, with more than two million instances, an average of 3.5 categories and 7.7 instances per image, and rich contextual information from diverse viewpoints for scene understanding. Its most frequent category, “person”, has almost 800,000 instances, whereas “hair dryer” has only about 600 [1,2].

Visual Genome [216], introduced in 2016, contains 108,077 images with over 3.8 million object instances across 1600 object categories, 2.8 million attributes, and 2.3 million pairwise relationships, together with region descriptions, scene graphs, and question–answer pairs, making it a comprehensive resource for scene understanding and relationship modeling [42]. Open Images [45], created by Google in 2017 and updated six times since, contains 9.2 million images with image-level labels, bounding boxes, and segmentation masks, including 16 million boxes for 600 categories on 1.9 million images (8.3 categories per image on average), visual relationship annotations such as “beer on the table,” and 2.8 million instance masks across 350 classes; it is the largest available detection dataset [1,2]. Finally, the ImageNet Large-Scale Visual Recognition Challenge (ILSVRC) [217], held annually from 2010 to 2017, provides over one million images in 1000 classification categories, and its detection task uses 200 manually selected categories with more than 500,000 images collected from ImageNet and Flickr [1,2].

### 6.2. Autonomous Driving Datasets

Autonomous driving datasets, mainly captured from a street-view camera perspective and often focused on pedestrians, provide the large-scale annotated real-world data needed to train models to recognize vehicles, infrastructure, and vulnerable road users [170]. They standardize evaluation for collision avoidance and navigation [218]; however, multi-sensor data capture is expensive, rare long-tail situations (e.g., adverse weather or unexpected objects) are hard to cover, and pedestrian detection is further complicated by heavy occlusion, darkness, and the variety of human physiques and actions [218]. Geographic and demographic bias can also impede real-world generalization, so the field is moving toward temporally richer data, stricter attribute annotations (e.g., degree of occlusion), and more diverse global environments [2]. Table 10 compares the key datasets discussed below with their primary annotations and the key features.

KITTI [219], recorded in Karlsruhe in 2012 by the Karlsruhe Institute of Technology and Toyota Technological Institute, was the first large-scale properly annotated benchmark with a complete autonomous driving sensor suite: high-resolution RGB and grayscale stereo cameras, a LiDAR with 100k points per frame, GPS/IMU measurements, object tracklets, and calibration data. Its 2D and 3D object detection benchmarks contain 7500 training and 7500 test images. BDD100K [220], released in 2017 by the University of California, Berkeley, is one of the largest and most diverse driving datasets, with 100,000 videos and image subsets of 100,000 and 10,000 images. It provides lane markings, pixel-wise semantic, instance and panoptic segmentation, pose labels, and box annotations for ten categories of common road objects (e.g., vehicles, buses, pedestrians, bicycles, trucks, and motorcycles) together with time-of-day and weather metadata, split 7:2:1 into training, test, and validation sets [221].

Cityscapes [222] targets semantic understanding of urban street scenes, with 5000 finely and 20,000 coarsely annotated images from 50 European cities across seasons, weather, and urban layouts. Its dense pixel-level semantic and instance annotations together with neighboring video frames, stereo views, and ego-vehicle data make it a premier benchmark for semantic, instance, and panoptic segmentation [223]. nuScenes [224] contains 1000 curated 20-s scenes with multimodal data and 3D annotations across diverse weather, lighting, and geographic conditions; its nuImages extension adds 93,000 images with instance masks, 2D boxes, and attributes for over 800,000 objects [225]. Active learning was used to target challenging scenarios and rare classes, and temporal context from past and future frames supports dynamic scene understanding [226,227,228].

The Waymo Open Dataset [229] offers synchronized high-resolution camera images and dense LiDAR point clouds with precise 3D bounding boxes across diverse urban scenarios, together with a robust metrics framework and TFRecord and Parquet formats. Its size demands substantial computational resources; in addition, sparse annotation of rare objects limits long-tail generalization [230] and its geographic focus may introduce bias. Nonetheless, it remains a cornerstone benchmark for 3D detection [231].

**Table 10:** Comparative analysis of key autonomous driving datasets.

Dataset (Year)	Sensor Modality	Size	Primary Annotations	Key Features/Use Case
KITTI (2012) [219]	Lidar, Stereo Cam, GPS/IMU	15k frames	3D/2D BBox, Tracklets	Pioneer dataset; complete sensor suite; General 3D perception
BDD100K (2017) [220]	Mono Camera	100k videos	2D BBox, Seg., Lanes	Time of day/weather metadata; 2D perception, scene parsing
Cityscapes (2016) [222]	Stereo Cam	5k fine + 20k coarse imgs	Dense Semantic & Instance Seg.	50 European cities; Pixel-wise urban scene understanding
nuScenes (2019) [224]	Lidar, Radar, 6x Cams	1k scenes	3D Cuboids, 2D BBox	Diverse weather; Temporal context; Multi-modal fusion
Waymo Open (2019) [229]	Lidar, 5x Cams	Large-scale	Precise 3D BBox	Diverse urban scenarios; Large-scale 3D detection
Caltech Ped. (2009) [232]	Mono Camera	250k frames	2D BBox (Peds)	Occluded, low-res pedestrians; Pedestrian detection benchmark
EuroCity Persons (2018) [233]	Mono Camera	47.3k images	2D BBox, Orientation	31 EU cities; Pedestrian/cyclist detection and orientation
iROADS (2014) [234]	Mono Camera	4656 frames	Vehicle BBox	7 adverse conditions; Vehicle detection in challenging weather
ONCE (2021) [235]	Lidar, Cams	1M scenes, 7M imgs	3D BBox	Massive scale; Self-supervised 3D perception
TU-DAT Dataset (2025) [236]	Camera and CCTV	12,351 frames	3D BBox	Analyzing road accidents

The Caltech Pedestrian Dataset [232], which is two orders of magnitude larger than earlier pedestrian datasets, contains richly annotated video from a moving vehicle with frequent occlusion, low-resolution pedestrians, and dynamic urban scenes. It revealed flaws in per-window evaluation metrics, leading to more accurate per-image measures [237], and showed that even state-of-the-art detectors such as the then-dominant HOG-based method could fail on small or partially occluded pedestrians, thereby motivating research into multi-frame analysis, contextual reasoning, and part-based models [238]. EuroCity Persons [233], collected by vehicle in 31 cities across 12 European countries by both day and night, contains over 238,200 person instances in more than 47,300 images, nearly an order of magnitude more than previous benchmarks, with over 211,200 orientation annotations for pedestrians, cyclists, and riders [237]. Benchmarking with Faster R-CNN, R-FCN, SSD, and YOLOv3 showed that data volume and diversity are pivotal, particularly for small or occluded persons [239]. Its updated version, EuroCity Persons 2.0 [240], recorded in 29 cities in 11 countries, supports person detection, tracking, and prediction in traffic [241].

iROADS [234] contains 4656 color frames captured from moving vehicles under seven adverse conditions, such as rainy day and night, snow, sun strokes, and tunnels, to test the robustness of vehicle and lane detection in Advanced Driver Assistance Systems (ADAS) [9]. Although it lacks ego-motion and stereo data, it targets the conditions in which perception is most likely to fail [242]. ONCE [235] provides one million LiDAR scenes and seven million camera images from 144 driving hours, a twenty-fold increase over nuScenes and Waymo, helping to support self- and semi-supervised 3D perception and address the long-tail problem; however, its size requires computing and storage resources that may be prohibitive for many institutions [243,244], and its initial benchmarks cover only 3D detection. Finally, TU-DAT [236], introduced in 2025, is a public benchmark for analyzing traffic accidents from a roadside camera perspective, with about 280 real and simulated videos annotated with vehicle trajectories and collision types. It captures aggressive pre-crash behaviors such as weaving and tailgating under daylight, night, fog, and rain, although simulated data may introduce a reality gap and roadside deployment raises privacy and edge latency challenges.

### 6.3. Remote Sensing (Aerial View) Datasets

**Figure 15:**
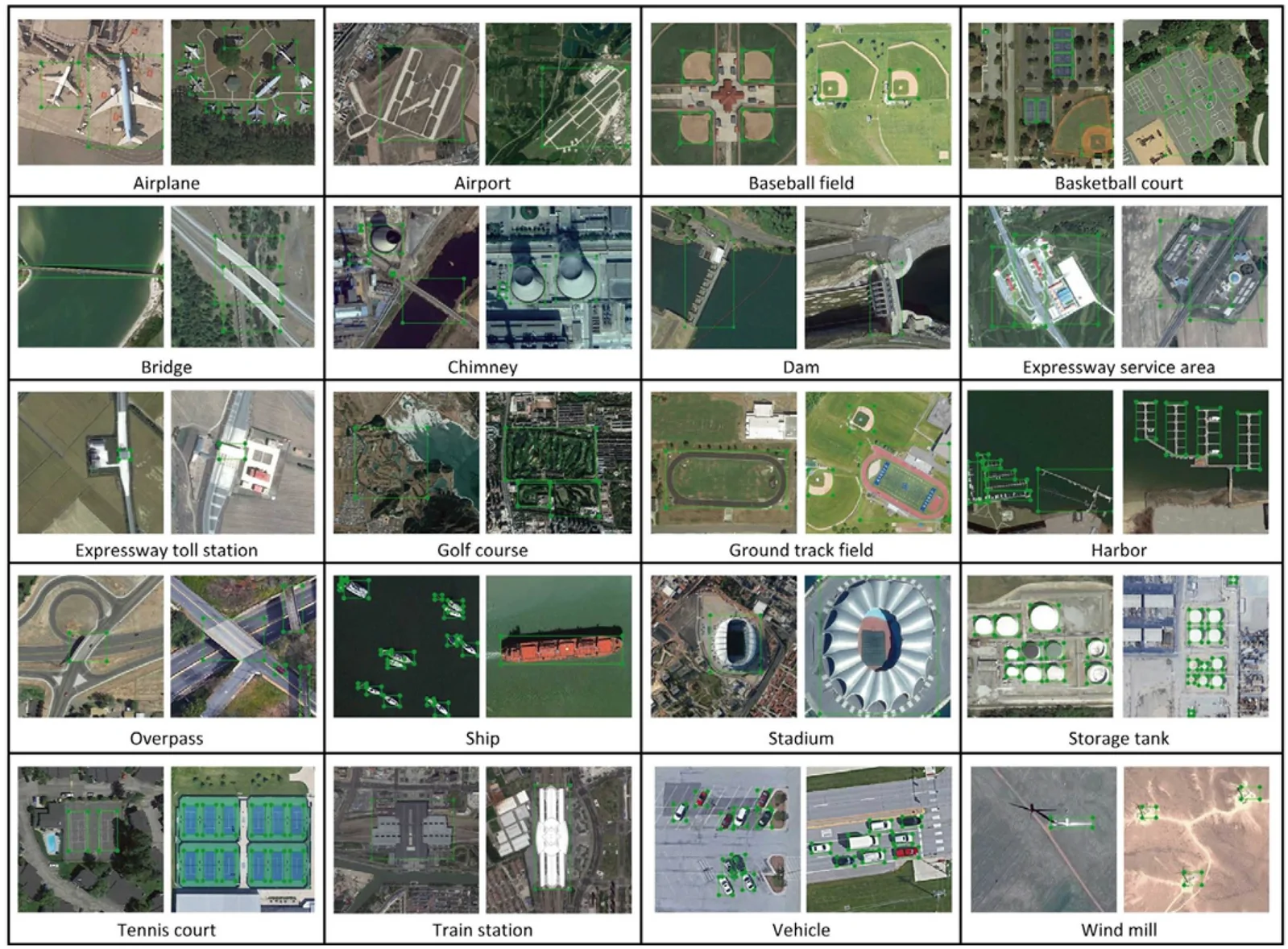
Example remote sensing datasets captured by drones and satellites in different imaging conditions, weather, seasons, and image quality [247].

Remote sensing datasets captured by satellites and unmanned aerial vehicles (UAVs) provide a top-down perspective for identifying and tracking vehicles, aircraft, ships, and infrastructure across vast and often inaccessible areas [245,246], thereby supporting urban planning, precision agriculture, disaster response, and national security. Figure 15 shows examples captured under different conditions [247]. Limitations include the high cost of capturing and storing high-resolution imagery, heavy computational demands, occlusion, extreme variation in object scale and orientation, and atmospheric conditions such as cloud cover [245,248].

DOTA-v2.0 [249] is compiled from multiple sources, sensors, and platforms, with images ranging from 800 × 800 to approximately 29,200 × 27,620 pixels. Expert annotators labeled 1.8 million object instances in 18 categories using oriented bounding boxes (OBBs), each with a difficulty score; this dataset is widely used in competitions such as LUAI 2021 [250]. xView [251] contains over one million objects in 60 classes across 1400 km^2^ of WorldView-3 imagery at 0.3 m per pixel, supporting few-shot learning and domain adaptation for disaster response and humanitarian efforts [252]. Its long-tail class distribution causes imbalance, while its resolution and geographic diversity pose computational and scale challenges [253]; its complex three-stage annotation process illustrates the cost of curating such data.

VEDAI [254] focuses on detecting small vehicles in unconstrained aerial scenes, with over 3700 annotated vehicles under varied orientation, lighting, occlusion, and background in both color and infrared bands at multiple resolutions. Baseline detectors achieved low recall, establishing VEDAI as a challenging benchmark that motivates multi-spectral fusion and context-aware small object detection [255]. DIOR [256] contains 23,463 optical remote sensing images with 192,472 instances in 20 categories, with large variations in object size, spatial resolution, imaging conditions, season, and weather that produce high inter-class similarity and intra-class variation [247]. Although it does not fully capture global diversity and some classes remain underrepresented, it is a strong benchmark for data-driven methods. The INRIA Aerial Image Labeling Dataset [257] tests generalization by training and testing on different cities (e.g., training on Chicago and testing on San Francisco or alpine towns in Austria), covering 810 km^2^ of 0.3 m imagery [258]. It is limited to binary building/non-building segmentation [259] and its test labels are withheld, so evaluation is possible only through an online portal [248]. Table 11 compares these remote sensing datasets below.

### 6.4. Face Detection Datasets

Face detection datasets are the cornerstone of training and benchmarking algorithms that locate human faces in images [260]. They have evolved from constrained laboratory collections to large “in-the-wild” benchmarks capturing occlusion, extreme illumination, diverse poses, and demographic variety; pioneering examples include Labeled Faces in the Wild (LFW) [261], WIDER FACE [262], and occlusion-specific collections such as MAFA and the Niqab dataset, which provide precise annotations of occlusion type and severity [263,264]. They serve security, biometric authentication, and augmented reality applications [265], but suffer from task specificity, biases related to age, ethnicity, and gender, privacy and consent concerns, uneven accessibility, and rapid obsolescence [266], motivating more diverse, fair, and ethically governed datasets [265,266]. Table 12 compares the key datasets.

WIDER FACE [262] is an order of magnitude larger than earlier face datasets, with rich annotations of extreme poses, substantial occlusion, and challenging lighting across numerous event categories, providing a realistic benchmark for surveillance and public space scenarios. Very small and heavily occluded faces remain difficult for current detectors [41], its event-based categorization may introduce bias, and the lack of identity labels limits its use for face recognition [266]. LFW [267] contains over 13,000 images of 5749 individuals captured under natural variations in pose, lighting, expression, background, and image quality [261], with predefined matched and mismatched pairs for consistent face verification benchmarking. However, its celebrity focus introduces demographic bias toward Western public figures, manual labels may contain inaccuracies, and its size limits training deep models from scratch [268].

**Figure 16:**
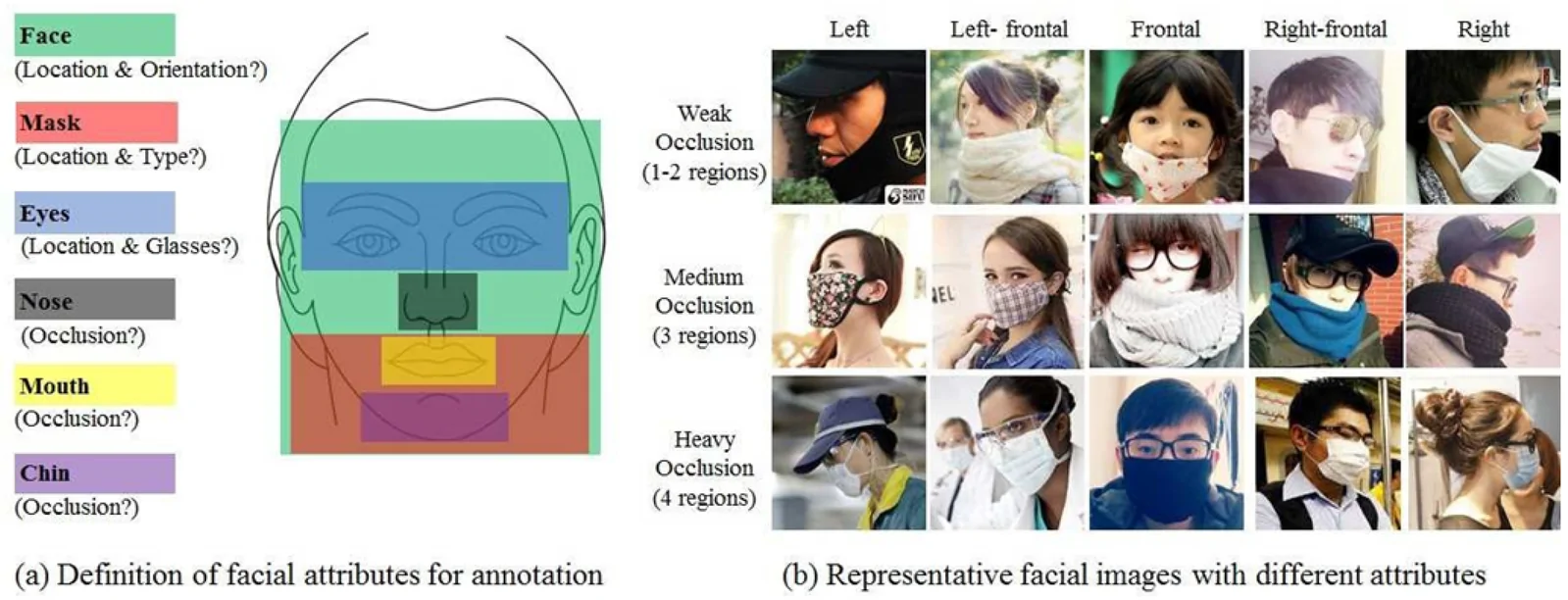
Definition of facial attributes and representative facial images in MAFA [263].

MAFA [263] addresses the detection of occluded faces, particularly those covered by masks or accessories such as hijabs or niqabs [264,269,270]. It contains over 30,000 internet images with more than 35,000 masked faces, each annotated with six attributes: face, eye, and mask locations, face orientation, occlusion degree (weak, medium, or heavy), and mask type (simple, complex, human body, or hybrid) (Figure 16a), yielding 60 combinations of orientation, occlusion degree, and mask type; Figure 16b shows representative images. Its cross-validated annotations make it a valuable benchmark for occlusion-robust face detection in public health and surveillance [271].

### 6.5. Medical Imaging Datasets

Medical imaging datasets provide annotated images from modalities such as X-ray, MRI, CT, and ultrasound for clinical tasks ranging from detecting tumors and fractures to segmenting organs and monitoring disease progression [272,273]. They enable reproducible benchmarking across institutions and the training of models that recognize subtle disease patterns, sometimes surpassing human accuracy in early cancer detection [274]. However, they face privacy restrictions on data sharing, costly expert annotation, and biases related to demographics, equipment, and disease representation. Larger, more diverse, and harmonized datasets along with federated learning to overcome data silos and rigorous validation are needed to translate AI into clinical practice [275,276]. Table 13 compares these datasets.

The Cancer Imaging Archive (TCIA) [277] is an open-access repository of CT, MRI, and PET images linked to clinical data across over 54 organ sites, supporting AI development, radiomics, and radiogenomics without the cost of new data collection [278]; however, differing acquisition protocols and incomplete clinical annotations can complicate analysis [279]. The Alzheimer’s Disease Neuroimaging Initiative (ADNI) [280] is a longitudinal multi-center study providing standardized MRI, PET, genetic, and fluid-biomarker data from controls and individuals with mild cognitive impairment (MCI) or Alzheimer’s disease (AD) [281]. It has enabled models predicting MCI-to-AD conversion and the validation of diagnostic tools [282,283], although its US/Canadian cohort and highly motivated volunteers may limit generalizability. The UK Biobank (UKB) [284] combines imaging, genetic, lifestyle, and health record data from over 500,000 participants in a longitudinal design, enabling large-scale epidemiology, multimodal AI, and biomarker discovery [285,286,287]; healthy volunteer bias and complex data wrangling are its main limitations [288].

The Open Access Series of Imaging Studies (OASIS) [289,290] provides freely available brain MRI, and in later versions, PET and clinical data focused on normal aging and AD, including longitudinal scans of cognitively normal and impaired individuals [291]. It is widely used to develop brain segmentation algorithms and AD biomarkers, although its predominantly older, Western, and right-handed volunteer cohort limits generalizability [292]. MIMIC-CXR [293,294] contains over 377,000 de-identified chest radiographs paired with free-text radiology reports from the Beth Israel Deaconess Medical Center, supporting automated pathology detection (e.g., pneumonia, pneumothorax) and report generation. Its single-institution retrospective origin may introduce bias, and labels extracted from reports can be noisy [295]; nevertheless, it underpins computer-aided diagnosis and triage tools [296].

The International Skin Imaging Collaboration (ISIC) datasets [297,298] contain tens of thousands of dermoscopic images with gold standard diagnoses and are the standard benchmark for automated skin cancer detection, driven by annual public challenges [299]. Limitations include that duplicate images across versions can inflate performance metrics, and class imbalance and label noise also remain concerns [300,301]. The Breast Cancer Digital Repository (BCDR) [302] provides digitized film and full-field digital mammograms with clinical data, which allows for evaluating the effect of image quality and combining image descriptors with clinical information [303]; however, its relatively small scale can constrain deep learning [304]. The Medical Segmentation Decathlon (MSD) [305] tests the generalizability of segmentation algorithms across ten tasks covering organs such as the brain, liver, prostate, and lungs in MRI and CT, a hypothesis validated by the winning nnU-Net framework. Per-task data requirements are modest, but scanner domain shift is not fully addressed [306]. MSD has shown that accurate models can be trained automatically, thereby accelerating clinical translation [307]. Finally, the Retinal Fundus Multi-disease Image Dataset (RFMiD) [308,309] annotates fundus images for 46 ocular conditions, including rare sight-threatening diseases, enabling more comprehensive screening tools. Images from three different fundus cameras introduce variability that models must handle [310].

### 6.6. Agricultural Image Datasets

Agricultural image datasets captured by drones, satellites, and ground-based sensors and annotated for crops, weeds, pests, diseases, and soil conditions underpin AI and computer vision models for precision agriculture [311], enabling scalable monitoring of plant health, targeted resource application, and early threat detection that increase yields while reducing water, pesticide, and fertilizer use [312]. Their limitations include costly annotation requiring agronomic expertise, a lack of standardization, and sensitivity to variation in lighting, growth stage, and weather that hinders generalization [313]; nonetheless, they are indispensable for food security, sustainable farming, and climate-resilient agriculture [314]. Table 14 shows that these datasets cover a wide diversity of tasks and species:

**Table 13:** Comprehensive comparative analysis of medical imaging datasets.

Dataset	Modality	Primary Focus	Scale	Annotations	Key Features/Advantages	Primary Limitation
TCIA [277]	CT, MRI, PET	Oncology	54+ organs; Numerous collections	Tumor segmentation, clinical data	Public, diverse cancer types, linked clinical data	Dataset-specific; varying annotation depth
ADNI [280]	MRI, PET	Neurology (Alzheimer’s)	3000+ subjects	Longitudinal segmentation, diagnosis	Longitudinal, multi-modal, well-established	Focused primarily on Alzheimer’s
UK Biobank [284]	MRI, CT, X-ray	Population Health	>100,000 participants	Organ segmentation, genetic, EHR	Unprecedented scale, genetic + imaging + EHR data	Access requires approval process
OASIS [290]	MRI	Neurology (Aging, Alzheimer’s)	1500+ subjects	Brain segmentation, clinical diagnosis	Curated for brain studies, publicly accessible	Smaller scale than UK Biobank
MIMIC-CXR [293,294]	X-ray (Chest)	Pulmonary	377,110 images	Free-text reports, labels from NLP	Large-scale, paired with EHR for NLP tasks	Labels automated from text, not pixel-wise
ISIC [297,298]	Dermoscopy	Dermatology	70,000+ images	Lesion segmentation, diagnosis	High-quality, standard benchmark for skin cancer	Mostly 2D images of lesions
BCDR [302]	Mammography	Breast Oncology	Thousands of cases	Lesion type, density, BIRADS	Focused on mammography, film and digital	Smaller than some modern benchmarks
MSD [305]	CT, MRI	Multi-organ Segmentation	Ten challenges	Pixel-wise segmentation	Tests generalizability across ten tasks	Designed as a challenge, not a unified db
RFMiD [309]	Fundus	Ophthalmology	3200 images	Unparalleled pathology diversity	Multi-source variability	Small in demographic scale

**Table 14:** Overview of agricultural object detection datasets (2025–2017).

Dataset Name	Year	Plant Name (English)	Plant Part	Task	Annotation	Images
CornLeafInfection [315]	2025	Maize	Leaf	Disease Detection	Bounding Boxes and segmentation	1308
PumkinDiseases [316,317]	2024	Pumpkin	Leaf	Disease Identification	Image Classes	2000
MFWD [318]	2024	Sorghum, Maize	Whole plant	Weed Detection	Img. Cls., BBox, Seg.	94,321
SorghumAphids [319,320]	2024	Sorghum	Leaf	Pest Detection	Segmentation Masks	54,742
PhenoBench [321]	2024	Sugar beet	Leaf, Whole plant	Weed Detection	Segmentation Masks	2179
SugarcanePlantCounting [322]	2024	Sugarcane	Cane, Fruit	Plant Counting	Bounding Boxes	3730
SoybeanNet [323]	2024	Soybean	Panicle, Fruit	Bean Counting	Seg. Masks, Points	196
CottonWeedDet3 [324]	2023	Cotton	Leaf	Weed Detection	Bounding Boxes	848
CropAndWeed [325]	2023	Various (Maize, Beet, etc.)	Leaf, Whole plant	Weed/Crop ID	Seg. Masks, BBox	8034
ImageWeeds [326]	2023	Various Weeds	Whole plant	Weed Identification	Bounding Boxes	3975
LucasVision [327]	2023	Various Crops	Ground cover	Plant Identification	Image Classes	16,946
RiceLeafDiseases [328]	2023	Rice	Leaf	Disease Detection	Image Classes	4684
RicePanicles [329]	2023	Rice	Panicle, Fruit	Panicle Detection	Bounding Boxes	2193
RumexLeaves [330]	2023	Broad-leaved Dock	Leaf	Plant Detection	Seg. Mask, BBox	809
SoyNet [331]	2023	Soybean	Leaf	Disease Detection	Image Classes	4500
TobaccoAerialDataset [332]	2023	Tobacco	Whole plant	Weed Detection	Segmentation Masks	1870
WE3DS [333]	2023	Various (Bean, Pea, etc.)	Leaf, Whole plant	Weed/Crop ID	Segmentation Masks	2568
YOLOWeeds [334]	2023	Various Weeds	Leaf	Weed Detection	Bounding Boxes	5648
CottonWeedID15 [335]	2022	Cotton	Leaf	Weed Identification	Image Classes	5187
ASDID [336]	2022	Soybean	Leaf	Disease Detection	Image Classes	9981
RoboWeedMap [337]	2022	Barley	Whole plant	Weed Detection	Bounding Boxes	1147
GlobalWheatHeadDetection [338,339]	2021	Wheat	Wheat Head	Plant Detection	Bounding Boxes	6422
DPA [340]	2020	Maize, Sugar beet	Whole plant	Plant Classification	Segmentation Masks	200
EarlyCropWeed [341]	2020	Tomato, Cotton	Leaf	Weed Detection	Image Classes	504
OpenWeedPhenotype [342]	2020	Various Weeds	Leaf	Weed Detection	BBox, Img. Cls.	7590
DeepWeeds [343]	2019	Various Weeds	Leaf	Weed Detection	Image Classes	17,509
DeepSeedling [344]	2019	Cotton	Seedlings	Seedling Detection	Bounding Boxes	5610
WeedGrowthState [345]	2018	Various Weeds	Leaf	Growth Estimation	Image Classes	12,165
MaizeDiseaseSymptoms [346]	2018	Maize	Leaf	Disease Detection	Other (Polyline)	18,222
PlantSeedlingClassification [347]	2017	Various	Seedlings	Seedling Classification	Image Classes	5659

CropAndWeed [325] and WE3DS [333] span many crop and weed species, MFWD [318] provides a massive scale of annotated imagery (about 94k images) [314], and DeepWeeds [343] and CottonWeedID15 [335] offer high-quality expert-verified annotations. Tasks range from seedling detection (DeepSeedling [344]) to in-season weed detection (PhenoBench [321]) and disease identification (ASDID [336], SoyNet [331]). While the public availability of many of these resources lowers the barrier to agricultural computer vision research [348], many datasets are small, which hinders training complex models and can lead to overfitting [349]. These datasets also show geographic and species bias towards major crops such as wheat, maize, and soybean; in addition, annotation types and quality vary dramatically, which complicates comparison and multi-dataset learning, and images captured under controlled conditions (e.g., CottonWeedID15 leaf images [335]) may not reflect real-field variability, creating a sim-to-real gap. Larger, more balanced, and more consistently annotated benchmarks that capture in situ conditions are still needed [349].

## 7. Evaluation Metrics for Object Detection

**Figure 17:**
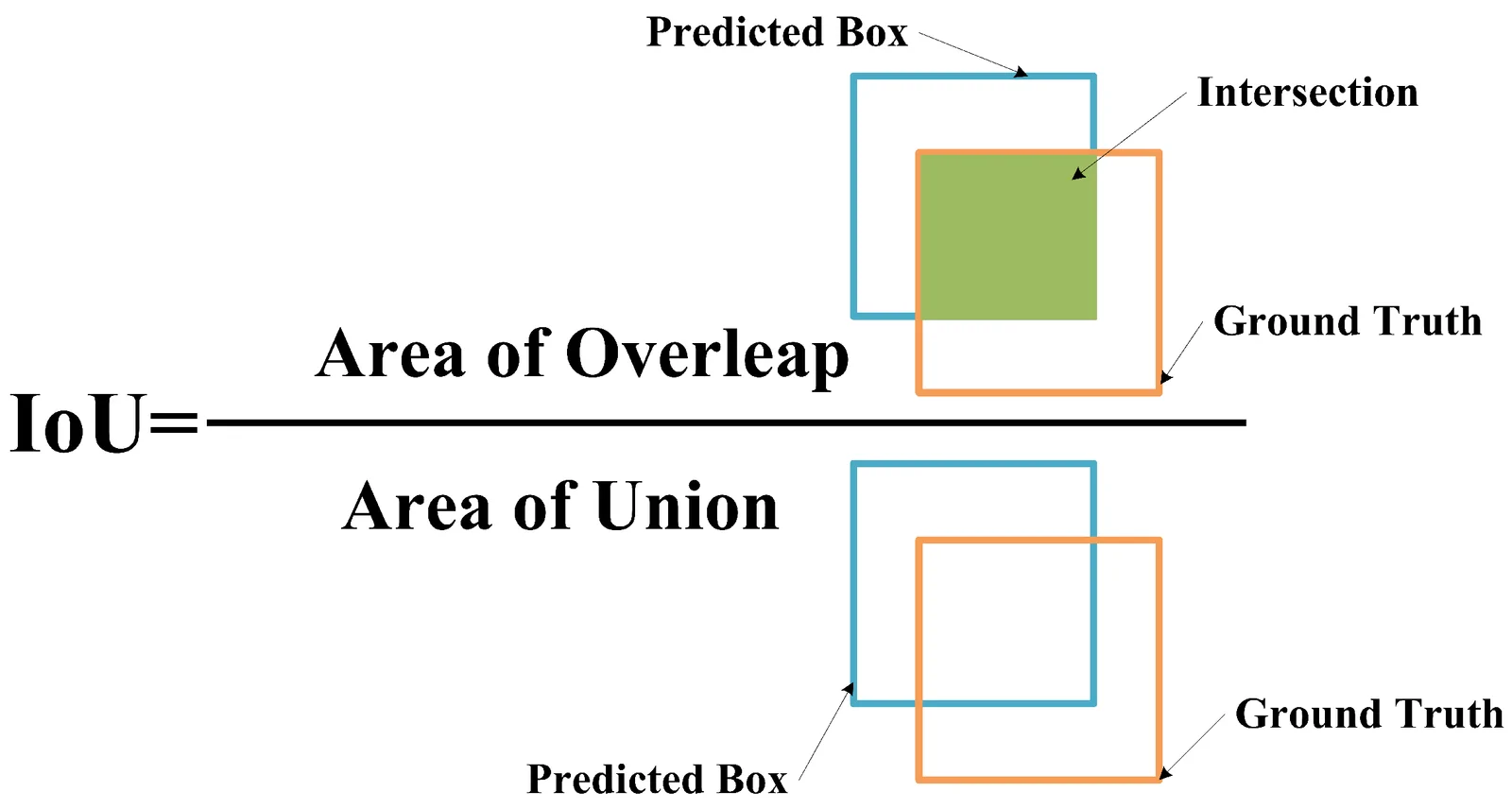
Demonstration of intersection over union (IoU).

Evaluation metrics quantify how well detectors identify and localize objects. Average precision (AP), the cornerstone of evaluation, is derived from the precision–recall curve and reflects the balance between correctness (precision) and completeness (recall) across confidence thresholds [260]. Because detection also requires accurate localization, the overlap between predicted and ground-truth boxes is measured by the intersection over union (IoU), with a common threshold of 0.5 defining a successful detection (Figure 17) [117,350]. For multi-class systems, the mean average precision (mAP) aggregates AP across categories; COCO-style AP averages results over stricter IoU thresholds (0.5–0.95) [104]. Class imbalance is addressed through F1 score optimization and adaptive thresholds, while real-world deployment also depends on efficiency metrics such as inference speed (FPS) and computational cost (FLOPs), particularly on edge devices and in real-time systems such as autonomous vehicles [82,170]. Confidence calibration, i.e., the agreement between a model’s self-reported certainty and its actual performance, is crucial for trust in safety-critical domains such as medical imaging [119,260]. This section presents fundamental metrics, IoU, the primary detection metrics AP and mAP, and specialized metrics such as object keypoint similarity (OKS) and panoptic quality (PQ).

Let TP, FP, and FN denote true positives, false positives, and false negatives, respectively. Precision (Equation (1)) is the fraction of predicted detections that are correct, indicating the model’s ability to identify only relevant objects, while recall (Equation (2)) is the fraction of ground-truth objects that are detected, indicating the model’s ability to find all relevant objects:

(1) $$\begin{array}{cc} \mathrm{Precision}(p) & =\frac{\mathrm{TP}}{\mathrm{TP}+\mathrm{FP}}, \end{array}$$

(2) $$\begin{array}{cc} \mathrm{Recall}(r) & =\frac{\mathrm{TP}}{\mathrm{TP}+\mathrm{FN}}. \end{array}$$

In object detection, true negatives (TN) are not well defined because background regions are not exhaustively annotated; consequently, metrics such as mAP, precision, and recall are preferred over overall accuracy, consistent with Padilla et al. [350]. When a single accuracy value is reported, it is computed from detections only, as in Equation (3):

(3) $$\begin{array}{cc} \mathrm{Accuracy} & =\frac{\mathrm{TP}}{\mathrm{TP}+\mathrm{FP}+\mathrm{FN}}. \end{array}$$

The F1 score (Equation (4)) is the harmonic mean of precision and recall. While mAP dominates benchmarks, F1 is favored in class-imbalanced or safety-critical settings, where minimizing missed detections (e.g., in medical imaging or surveillance) and limiting false alarms (e.g., in autonomous driving) must be balanced; it is often computed at the optimal confidence threshold, with class-wise averaging (macro-F1) for multi-class analysis.

(4) $$F_{1}=2\times \frac{\mathrm{Precision}\cdot \mathrm{Recall}}{\mathrm{Precision}+\mathrm{Recall}}.$$

In dense scenes such as crowded pedestrian scenes or satellite imagery, precision–recall curves may inadequately capture performance. The miss rate (MR) and false positives per image (FPPI) can bbe used instead, often combined in an MR–FPPI curve. The MR (Equation (5)) is the proportion of undetected ground-truth objects, while FPPI (Equation (6)) is the average number of false detections per image over $N_{\mathrm{images}}$ images:

(5) $$\begin{array}{cc} \mathrm{Miss}\;\mathrm{Rate}(\mathrm{MR}) & =\frac{\mathrm{FN}}{\mathrm{TP}+\mathrm{FN}}=1-\mathrm{Recall}, \end{array}$$

(6) $$\begin{array}{cc} \mathrm{False}\;\mathrm{Positives}\;\mathrm{Per}\;\mathrm{Image}\;\left(\mathrm{FPPI}\right) & =\frac{\mathrm{FP}}{N_{\mathrm{images}}}. \end{array}$$

IoU quantifies the spatial overlap between a predicted bounding box and its ground-truth annotation as the ratio of their intersection area to their union area (Figure 17 and Equation (7)) [213,350]:

(7) $$\mathrm{IoU}=\frac{\mathrm{Area}(B_{\mathrm{pred}}\cap B_{\mathrm{true}})}{\mathrm{Area}(B_{\mathrm{pred}}\cup B_{\mathrm{true}})}.$$

A detection is classified as a TP if its IoU exceeds a threshold τ, commonly 0.5 in benchmarks such as PASCAL VOC, while stricter thresholds (e.g., τ=0.75) are used in COCO to evaluate high-precision localization. IoU does not account for object orientation or shape (e.g., rotated or irregular objects) and is sensitive to minor positional deviations for small objects, but its simplicity, interpretability, and widespread adoption make it the foundation for AP and mAP.

AP is the primary metric for a single class, summarizing the precision–recall curve into a single number that reflects both accurate (high precision) and complete (high recall) detection (Equation (8)). The mean average precision (mAP) averages AP over all *N* classes (Equation (9)), where ${\mathrm{AP}}_{i}$ is the AP of the *i*-th class:

(8) $$\begin{array}{cc} \mathrm{AP} & =\int_{0}^{1}p\left(r\right)\;dr, \end{array}$$

(9) $$\begin{array}{cc} \mathrm{mAP} & =\frac{1}{N}\sum_{i=1}^{N}{\mathrm{AP}}_{i}. \end{array}$$

While the AP evaluates how well the detector balances precision and recall for one class while minimizing FP and FN, the mAP standardizes multi-class evaluation and enables fair comparison between detectors. For example, in a dataset with four classes for which the AP values are $AP_{cats}=0.85$, $AP_{human}=0.87$, $AP_{dogs}=0.89$, and $AP_{car}=0.92$, the mAP is computed as in Equation (10):

(10) $$mAP=\frac{0.85+0.87+0.89+0.92}{4}=0.88.$$

Specialized tasks require more nuanced metrics; for keypoint detection tasks such as human pose estimation, IoU is poorly suited to evaluating individual keypoints, so the object keypoint similarity (OKS) is the canonical metric (Equation (11)):

(11) $$OKS=\frac{\sum_{i}\exp\left(-\frac{d_{i}^{2}}{2s^{2}\kappa_{i}^{2}}\right)\cdot \delta \left(v_{i}>0\right)}{\sum_{i}\delta \left(v_{i}>0\right)}$$

where $d_{i}$ is the Euclidean distance (in pixels) between the *i*-th predicted keypoint and its ground truth, *s* is the object scale, typically $\sqrt{wh}$ of the bounding box for a person, $\kappa_{i}$ is a dataset-dependent per-keypoint constant derived from annotation uncertainty (e.g., small for eyes and ears and larger for shoulders and hips), and $\delta (v_{i}>0)$ equals 1 if the ground-truth keypoint is labeled and visible and equals 0 otherwise, so non-annotated keypoints do not contribute. The exponential term is a Gaussian that approaches 1 for accurate predictions and penalizes deviations relative to the object size and keypoint uncertainty [46]. The OKS lies between 0 and 1, and performance is reported as mAP over OKS thresholds from 0.5 to 0.95 in steps of 0.05 (${\mathrm{mAP}}^{\mathrm{OKS}}$).

Panoptic segmentation, which segments both countable “things” and amorphous “stuff”, is evaluated with the panoptic quality (PQ) metric (Equation (12)), which combines segmentation and recognition quality into a single score:

(12) $$\mathrm{PQ}=\frac{\sum_{(p,g)\in TP}\mathrm{IoU}\left(p,g\right)}{\left|TP\right|+\frac{1}{2}\left|FP\right|+\frac{1}{2}\left|FN\right|}.$$

PQ decomposes into the product of segmentation quality (SQ, Equation (13)), the average IoU of matched segments, which measures how well predicted masks align with ground-truth masks, and detection quality (DQ, Equation (14)), which is identical to the F1 score and measures recognition accuracy:

(13) $$\begin{array}{cc} \mathrm{SQ} & =\frac{\sum_{(p,g)\in TP}\mathrm{IoU}\left(p,g\right)}{\left|TP\right|}, \end{array}$$

(14) $$\begin{array}{cc} \mathrm{DQ} & =\frac{\left|TP\right|}{\left|TP\right|+\frac{1}{2}\left|FP\right|+\frac{1}{2}\left|FN\right|}. \end{array}$$

## 8. Applications of Object Detection

Object detection has moved from benchmark evaluation to operational deployment across a wide range of domains. Across these domains, the field is shifting from closed-set toward open-vocabulary and language-conditioned detection, from RGB-only input toward multimodal sensor fusion (LiDAR, radar, thermal, and event sensors), from server-grade GPUs toward edge platforms (driving quantization, pruning, distillation, and hardware-aware design), and from mAP-only evaluation toward calibration, domain-shift robustness, and failure rate and fairness analysis, with the latter supported by application-specific benchmarks because generic benchmarks such as MS-COCO do not represent the long-tail distributions and sensor characteristics of real deployments.

In transportation, detection is indispensable for autonomous vehicles [351], which must identify pedestrians, vehicles, cyclists, traffic signs, and other obstacles in real time in order to navigate safely. Traffic monitoring systems use detection to analyze flow patterns, identify congestion, and optimize signal control [352,353,354]. Robustness under adverse weather, low illumination, and sensor degradation is a key requirement, motivating the fusion of cameras, LiDAR, and radar, and cooperative multi-agent systems that extend detection to fleets of unmanned vehicles, where inter-agent perception and obstacle detection are critical [355,356]. A similar demand for reliability under uncontrolled conditions appears in agriculture, where computer vision has enabled precision farming at scale [357,358]. Detection algorithms count crops, assess plant health, and identify pests and diseases [359,360], helping farmers to make informed decisions on irrigation, fertilization, and pest control while reducing input costs and environmental impact [361]. Because deployment typically occurs on UAVs and ground robots under variable lighting, foliage occlusion, and limited onboard compute, this domain favors lightweight single-stage detectors, echoing the edge deployment trend noted above.

The defense sector places even stricter constraints on latency and robustness. Detection supports surveillance, reconnaissance, and target identification by identifying potential threats, tracking targets, and providing real-time intelligence [362], while UAV-mounted systems support search and rescue, disaster relief, and border patrol [363]. The principal challenges here are cluttered and camouflaged scenes, low-contrast infrared imagery, and strict latency requirements for time-critical decisions. In contrast, industrial automation operates under controlled conditions but demands repeatability and integration with existing control infrastructure. Automated inspection systems identify surface defects, verify assembly quality, and reduce material waste [364], while tracking systems optimize production lines by monitoring the movement of materials and equipment [365,366,367]. In both defense and industry, the emphasis is on reliability and seamless integration rather than peak benchmark accuracy.

**Figure 18:**
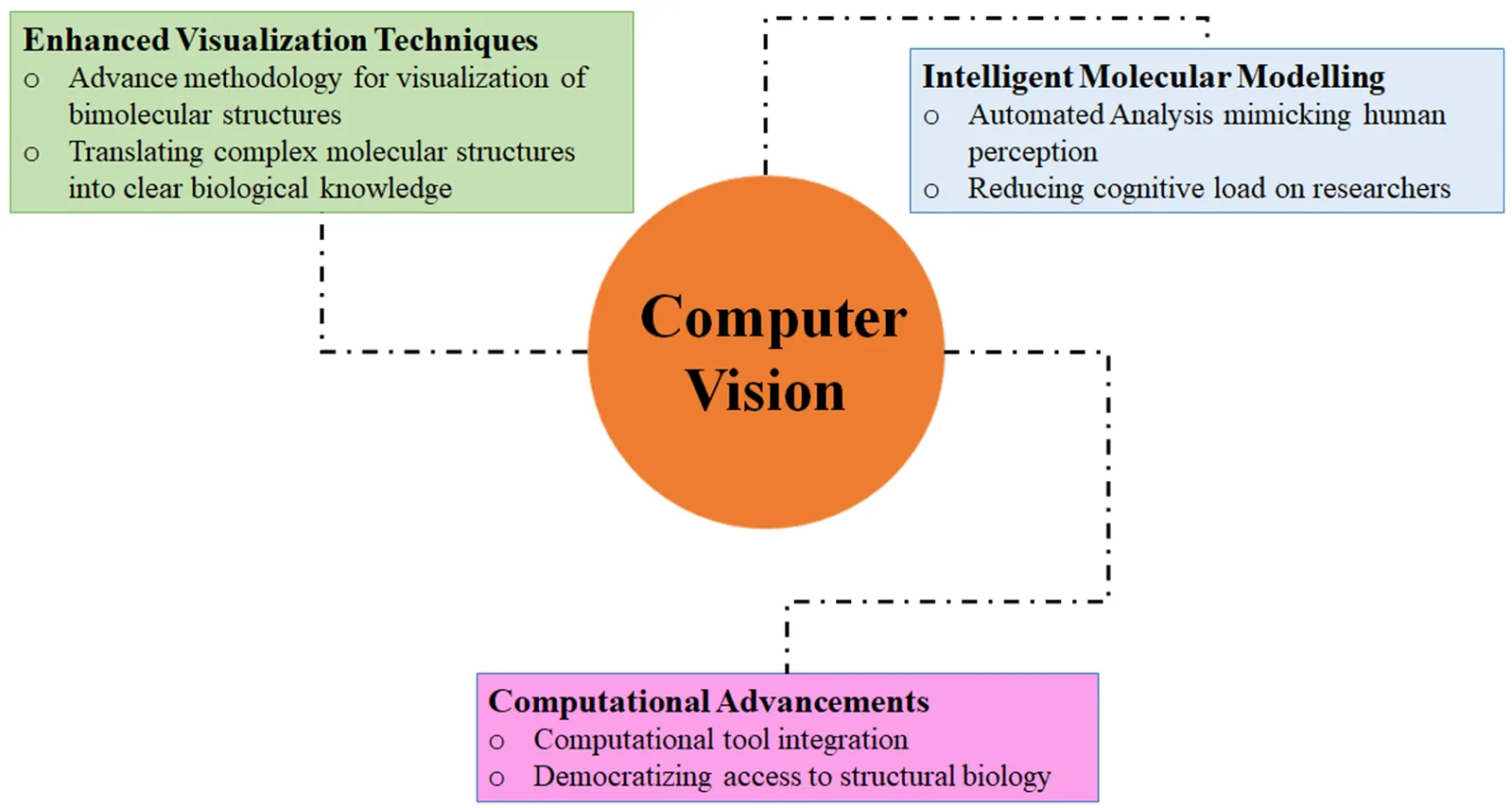
Applications of computer vision in structural biological research.

Healthcare represents a different regime altogether, in which the cost of error is high, and interpretability is essential. Detection algorithms assist medical image analysis, including tumor identification in radiographs, lesion detection in MRI and CT, and abnormality screening across modalities [368,369,370,371], thereby supporting patient monitoring, gait analysis, and surgical assistance [372]. Medical deployment imposes strict requirements on sensitivity, interpretability, and regulatory compliance. Beyond clinical imaging, the same detection principles transfer to structural biology, where object detection techniques are applied to localize and classify macromolecular particles in cryo-electron microscopy images, identify and track individual molecules in super-resolution microscopy, and automate the annotation of cellular structures in large-scale imaging datasets. For example, automated particle picking in cryo-electron microscopy (cryo-EM) localizes individual particles in noisy micrographs, a task analogous to detecting objects in natural images; this has the effect of reducing manual effort and improving throughput in three-dimensional structure reconstruction tasks [373]. Similar transfer has been demonstrated in cell detection in fluorescence microscopy, defect identification in materials science, and object localization in astronomical surveys (Figure 18). These examples show that detection architectures developed for natural images generalize to scientific imaging domains with minimal adaptation.

Finally, detection contributes to environmental monitoring and education, two domains where accessibility and cost-efficiency matter more than peak accuracy. Detection models track wildlife populations, monitor deforestation, and identify pollution sources, providing data for conservation and environmental management [374,375]. In education, detection enables interactive learning environments in which students engage with virtual objects and concepts [376,377]. Taken together, these seven domains illustrate that object detection is no longer confined to benchmark datasets but is embedded in operational systems across transportation, agriculture, defense, industry, healthcare, science, the environment, and education, with each domain imposing its own combination of accuracy, latency, robustness, and interpretability requirements.

## 9. Discussion and Future Directions

The evolution of object detection from handcrafted feature extractors to deep convolutional networks, transformer-based architectures, and vision–language models reflects a broader shift toward generalist data-driven perception. Nevertheless, several interconnected challenges still constrain generalization, reliability, and deployment efficiency across differing sensor platforms. This section synthesizes these challenges across detector families and outlines balanced directions for future research.

### 9.1. Persistent Challenges Across Detector Families

A recurring limitation is data dependence: deep detectors learn passively from observed patterns, meaning that they inherit the coverage gaps and biases of their training datasets [378]. Benchmarks such as MS-COCO under-represent the long tail of real-world objects, occlusions, and intra-class variation, producing models that degrade on rare or novel instances [379]. A second barrier is computational cost: although lightweight YOLO variants have broadened real-time accessibility, many high-accuracy transformer- and LLM-based detectors still require multi-GPU infrastructure, limiting deployment on edge sensors [380]. A third challenge is annotation dependence: supervised detection requires large-scale bounding box annotations, which are expensive and prone to label noise, particularly in specialized domains such as medical imaging and remote sensing [381]. Fourth, open-vocabulary and multimodal perception introduces new challenges in spatial precision, training convergence, and inference latency [382]. Finally, reliability and calibration remain under-addressed: most evaluations report aggregate accuracy without quantifying uncertainty, calibration error, or failure modes, which limits trust for safety-critical deployments [350]. Table 15 maps each challenge to the detector families it affects most and the limitations that remain, distinguishing relatively mature problems from genuinely open ones.

### 9.2. Future Directions

Building on these gaps, we identify five balanced directions for future research, each corresponding to one of the research gaps identified in the Abstract and Introduction.

#### 9.2.1. Lightweight Edge Deployment

Lightweight YOLO variants (YOLOv8-nano, YOLOE) have democratized real-time inference, but many high-accuracy systems based on transformers and LLMs still require costly multi-GPU configurations, limiting accessibility and delaying deployment on resource-constrained devices [380]. Future work should balance accuracy (mAP) with deployment efficiency (FPS) on embedded sensor platforms through quantization-aware training, structured pruning, knowledge distillation from large teachers to compact students, and hardware-aware neural architecture search that jointly optimizes latency, memory, and energy. Standardized benchmarks should report accuracy–efficiency trade-offs on representative edge hardware (e.g., NVIDIA Jetson, Google Coral, mobile NPUs) rather than on server-grade GPUs alone.

#### 9.2.2. Sensor Fusion and Multimodal Perception

Most detectors operate on RGB input alone, yet safety-critical systems increasingly rely on complementary modalities such as LiDAR, radar, thermal infrared, and event cameras [383]. Sensor fusion introduces challenges in temporal synchronization, spatial calibration, and heterogeneous feature alignment, and is under-represented in benchmark-driven research. Future work should develop unified architectures that use cross-modal attention and joint representation learning to fuse multimodal streams natively rather than through late-stage post-processing; benchmarks should evaluate robustness under adverse weather, sensor degradation, and modality dropout, conditions in which single-modality detectors fail silently. This is essential for autonomous driving, industrial inspection, and defense applications, where sensor redundancy provides the only reliable path to safety.

#### 9.2.3. Bias, Fairness, and Generalization

Because public datasets under-represent both the long tail of real-world objects and demographic diversity, trained models inherit and can amplify these biases, producing uneven performance across populations and environments [108]. This has direct societal consequences in face detection, medical imaging, and surveillance. Future work should prioritize automatic and objective data exploration, label-efficient learning (few-shot, weakly supervised, and self-supervised), and fairness-aware evaluation that reports performance disparities across subgroups rather than aggregate accuracy alone. Synthetic data from generative models and simulators can increase diversity without exhaustive annotation, and active learning can prioritize the most informative samples for labeling. Addressing bias is not merely an ethical imperative but a technical prerequisite for deployment in varied real-world environments.

#### 9.2.4. Reliability for Safety-Critical Applications

Deployment in autonomous driving, medical diagnostics, and industrial automation demands quantified reliability, not just high average precision. Three aspects deserve particular attention: first, uncertainty quantification means that models should express calibrated confidence using techniques such as Monte Carlo dropout, deep ensembles, or evidential learning, allowing downstream systems to defer to human operators when uncertainty is high; second, confidence calibration means that predicted confidence must align with actual correctness to support human oversight and regulatory compliance, compensating for the overconfidence of most detectors on out-of-distribution inputs; third, domain shift and failure analysis requires that detectors degrade gracefully under distribution shift (new sensors, weather, or geographies) and expose interpretable failure modes rather than keeping errors hidden. Evaluation protocols should report calibration error, out-of-distribution detection performance, and failure rate metrics alongside mAP and IoU [350], which requires collaboration between the computer vision, control, and safety engineering communities.

#### 9.2.5. Vision–Language and Open-Vocabulary Integration

Vision–language models such as Grounding DINO, YOLO-World, and Florence-2 have demonstrated strong zero-shot generalization and open-vocabulary detection by leveraging natural language reasoning [382,384]; this is one promising direction among several for extending detection beyond closed-set categories. Remaining challenges include fine-grained spatial localization, slow training convergence, hallucination of non-existent objects, and high inference cost that limits edge deployment. Future work should ground language priors in precise spatial outputs, reduce computational overhead through efficient cross-modal attention and token pruning, and validate these models on domain-specific sensor data rather than general web imagery [206]. The effect of natural language prompts on model behavior under distribution shift also remains poorly understood and deserving of attention.

### 9.3. Synthesis and Outlook

Taken together, these five directions reflect a transition from benchmark-centric accuracy towards deployment-centric reliability. The next generation of detectors will be judged not only on mAP and FPS but on their ability to operate on different sensor platforms, generalize across domains, quantify their own uncertainty, and treat fairness as a first-class design objective. These directions are interdependent: lightweight architectures enable broader sensor deployment, sensor fusion improves robustness under adverse conditions, fairness-aware training mitigates dataset bias, reliability metrics provide evidence for safety certification, and vision–language integration expands the range of detectable concepts. We anticipate that object detection will evolve from a task-specific perception component into a general-purpose sensor-aware reasoning system embedded across autonomous driving, industrial automation, medical diagnostics, and scientific discovery.

## 10. Conclusions

This review has explored object detection algorithms over the last thirty years, from traditional algorithms to recent LLM-based models. It reviews and analyzes deep learning-based detectors, including single-stage, two-stage, transformer-based, and LLM-based detectors; analyzes object detection datasets and benchmarks according to their usability, together with the evaluation metrics of object detection algorithms; and tracks changes in research trends and future applications. By analyzing existing approaches, identifying research gaps and shifting research trends, and suggesting future developments, this review illuminates the opportunities and challenges in object detection.

## Figures and Tables

**Table 1:** Comparison of traditional object detection methods.

Method	Advantages	Disadvantages	Complexity	Applications
SIFT [52]	Robust to scale/rotation changes; strong feature matching	Slow; sensitive to illumination shifts	High	Object recognition, robotics vision
Cascade [58]	Real-time detection; efficient with integral image; good for frontal faces	Poor with non-frontal views and occlusions; sensitive to lighting and pose variation	Low	Face detection and real-time surveillance
HoG [63]	Effective for edge-based objects; lighting-invariant	Poor for textured/cluttered scenes	Medium	Pedestrian detection, vehicle recognition
DPM [67]	Handles occlusion; shape-invariant	Needs large datasets; slow inference	Very High	Face detection, cluttered scenes
SURF [71]	Faster than SIFT; decent accuracy	Unstable under extreme lighting/rotation	Medium	Image retrieval, 3D reconstruction
ORB [74]	Very fast; rotation-invariant; binary descriptor enables efficient matching	Sensitive to large scale changes; less robust than SIFT/SURF under heavy blur	Low	Real-time SLAM, mobile vision, augmented reality

**Table 2:** Comparison Between AlexNet, VGG-16, GoogLeNet, and ResNet-50, as presented in [2].

Model	Year	Layers	Parameters (Millions)	Top Accuracy (%)
AlexNet	2012	7	62.4	63.3
VGG-16	2014	16	138.4	73
GoogLeNet	2014	22	6.7	-
ResNet-50	2015	50	25.6	76

**Table 3:** Comparison of single-stage vs. two-stage object detectors.

Aspect	Single-Stage Detectors	Two-Stage Detectors
Speed	High (30–150 FPS)	Low to moderate (5–15 FPS)
Accuracy	Moderate (50–60 mAP on COCO)	High (60–70 mAP on COCO)
Complexity	Simple (single forward pass)	Complex (region proposals + refinement)
Use Case	Real-time applications (video, edge devices)	High-precision offline tasks (medical imaging, research)
Small Objects	Struggles due to coarse features	Better handles small/occluded objects

**Table 4:** Comparison of YOLO object detection algorithms.

Model	Year	Architecture	Key Improvements	Output Head	Anchor	Features
YOLOv1	2015	GoogLeNet	Unified detection, real-time speed	Fully connected	No	Single CNN for detection
YOLOv2	2016	Darknet-19	BatchNorm, high-res classifier, anchor boxes	Convolutional	Yes	Dimension clustering, fine-grained features
YOLOv3	2018	Darknet-53	Residual blocks, multi-scale prediction	Convolutional	Yes	Three-scale prediction, better backbone
YOLOv4	2020	CSPDarknet53	Weighted residual connections, PANet	Convolutional	Yes	Mish activation, SPP, SAM, Mosaic augmentation
YOLOv5	2020	Custom PyTorch	Light-weight, flexible deployment	Convolutional	Yes	Auto-learning bounding boxes, faster training
YOLOR	2021	Hybrid CNN	Implicit + explicit feature representation	Convolutional	Yes	Unified representation learning
YOLOX	2021	CSPDarknet + SimOTA	Decoupled head, anchor-free	Decoupled Head	No	Strong baseline, SimOTA label assignment
YOLOv6	2022	EfficientRep	Optimized for edge devices	Decoupled Head	No	SimOTA, higher FPS
YOLOv7	2022	E-ELAN	Extended efficient layer aggregation	Decoupled Head	Yes	Model scaling, multi-task support
YOLOv8	2023	Custom (C2f)	Modular, unified task support	Decoupled Head	No	Instance segmentation, pose estimation
YOLOv9	2024	GELAN+PGI	Programmable Gradient Information (PGI), Generalized ELAN (GELAN), information bottleneck	Decoupled Head	No	Sparse sampling, high accuracy
YOLOv10	2024	Enhanced C2f	Re-parameterized ELAN	Decoupled Head	No	Multi-head attention, inference stability
YOLOv11	2024	Improved C2f + Sparse Attention	Lightweight generalist model	Decoupled Head	No	Enhanced gradient flow, real-time optimization
YOLOv12	2025	R-ELAN + Area Attention	Attention-centric design, Area Attention, R-ELAN, FlashAttention	Decoupled Head + NMS-free	No	Joint detection, segmentation, and pose estimation

**Table 5:** Comparative analysis of transformer-based object detection and YOLO object detector.

Model	Architecture	Backbone	Detection Approach	Key Features	Attention Mechanism	Speed vs. Accuracy	Strengths	Weaknesses	Use Cases
ViT	Single-stage	Vision Transformer	Query-based	Uses ViT backbone; often paired with detection heads (e.g., ViTDet)	Global self-attention	Moderate speed, High accuracy	Strong global context modeling; scalable to large datasets	Computationally heavy; requires pretraining	High-accuracy detection tasks
DETR	Single-stage	CNN + Transformer	Query-based (set prediction)	End-to-end; anchor-free; bipartite matching loss	Encoder-decoder cross-attention	Slow training, Moderate inference	Simplifies pipeline; handles occlusions well	Slow convergence; high memory usage	General-purpose detection
SMCA DETR	Single-stage	CNN + Transformer	Query-based	Spatial-Modulated Co-Attention to focus on likely regions early	Multi-scale conditional attention	Faster convergence than DETR	Reduces training time; improves spatial reasoning	Still slower than YOLO/RetinaNet	Real-time with better accuracy
Anchor DETR	Single-stage	CNN + Transformer	Anchor-guided queries	Anchor-based object query initialization; improves convergence	Anchor-based cross-attention	Faster convergence than DETR	Better localization; efficient query design	Limited to predefined anchor layouts	Balanced speed/accuracy tasks
Swin Transformer	Single-stage	Swin Transformer	Hierarchical query-based	Shifted window attention; multi-scale feature representation	Local + shifted window attention	High accuracy, Moderate speed	Scales efficiently; fine-grained feature capture	Complex architecture; high compute	High-resolution detection
Deformable DETR	Single-stage	CNN + Transformer	Deformable attention	Sparse sampling around reference points; multi-scale feature fusion	Deformable attention	Faster than DETR	Efficient attention; good for small objects	More complex attention implementation	Scene-heavy or crowded environments
YOLO Family	Single-stage	CNN (DarkNet, etc.)	Anchor-based	Grid-based predictions; multi-scale feature fusion (e.g., FPN in YOLOv3+)	CNN-only (v1–v10); attention-based (v11: C2PSA; v12: Area Attention)	Very fast, Lower accuracy	Real-time performance; lightweight variants (e.g., YOLOv8-nano)	Struggles with small objects; anchor bias	Real-time applications (video, drones)

**Table 8:** Comparison between single-stage, two-stage, transformer-based, and LLM-based object detectors.

Feature	Single-Stage Detectors	Two-Stage Detectors	Transformer-Based Detectors	LLM-Based Detectors
Architecture	Single CNN pass; direct regression to bounding boxes and classes.	Stage 1: Region Proposal Network (RPN); Stage 2: Classification and Refinement.	Encoder–decoder transformer; uses self-attention to capture global context.	Vision model (often transformer) + language model; Uses text prompts for zero-shot detection.
Detection Speed	Fastest in real-time. Inference is a single step.	Slowest (high latency). Two sequential steps are required.	Highly variable (initially slow, but modern variants are real-time).	Generally slower than SSD/modern T-detectors due to language model overhead.
Accuracy (mAP)	High, but historically lower than TSDs. The gap has been significantly narrowed by recent YOLO variants.	Highest (Traditionally the state-of-the-art for precision). Refinement boosts accuracy.	Very high (competitive with TSDs). Excels with global context.	High in zero-shot/open-vocabulary tasks; accuracy varies on specific prompts.
Complexity	Low to moderate. Simpler pipeline (no RoI Pooling/NMS-free in new versions).	High. Complex multi-component pipeline (RPN, RoI Align, NMS).	High. Transformer architecture is parameter-heavy; slow convergence in training.	Highest. Involves two complex large models (vision + language).
Small Object Effectiveness	Moderate. Struggles due to dense sampling and limited resolution in the final detection layers.	High. The two-stage refinement process and use of multi-scale features (FPN) significantly boost small object accuracy.	Moderate to high. Early models struggled, but modern variants with multi-scale features and higher resolution handle small objects well.	Potentially high (context-dependent). Can use language to infer the location of small, hard-to-see objects based on scene context.
Primary Use Case	Real-time systems, video surveillance, autonomous driving (for speed).	Offline analysis, medical imaging, quality inspection (where accuracy is paramount).	General-purpose OD, scenarios needing a strong global context (e.g., crowded scenes).	Open-vocabulary detection, zero-shot learning, language-guided search.

**Table 9:** Comparison of generic object datasets.

Dataset	Classes	Images	Applications	Advantages	Disadvantages	Contributions
Caltech-101 (2003) [214]	102	9144	Generic Object Recognition	Early dataset moving beyond iconic views to natural environments	Limited to one object per image and not designed for modern detection tasks	Early efforts to include object categories in natural environments, primarily for classification
PASCAL VOC (2007/2012) [44]	20	11,530	Generic Object Detection	Relatively small and convenient for benchmarking, and established standard evaluation metrics (mAP)	Small size by modern standards, Limited to 20 classes, superseded by larger datasets	The foundational dataset that set the benchmark for object detection and evaluation metrics (27,450 annotated object instances)
Caltech-256 (2006) [215]	257	30,307	Generic Object Recognition	Early dataset expanding object categories in natural environments	Limited to one object per image and not designed for modern detection tasks	Extended Caltech-101 with more categories and improved dataset construction
MS COCO (2014) [46]	91	328,000	Generic Object Detection	High object density in natural, contextual environments with rich annotations	Basic object categories lack fine granularity, and the annotation process is complex and expensive	Established a benchmark emphasizing context and segmentation beyond simple bounding boxes
Visual Genome (2016) [216]	76,340	108,000	Scene Understanding	Dense annotations of objects, attributes, relationships, and QA pairs with a massive vocabulary of object classes	Annotations may be noisy/inconsistent due to crowdsourcing, where not all images are fully annotated	Introduced dense scene graphs linking visual data with structured semantics
Open Images (2017) [45]	600	9,178,275	Large-Scale Object Detection	Extremely large scale (9M+ images), including relationship annotations that provide negative labels	Initially lacked segmentation masks (added later), where severe object classes are imbalanced due to web sourcing	One of the largest datasets, emphasizing real-world diversity and relationship annotations

**Table 11:** Comparative analysis of key remote sensing (aerial view) datasets for object detection.

Dataset	Scale	Resolution	Key Features and Advantages	Limitations
DOTA (v2.0) [249]	1.8M instances	800 × 800 to ∼29,200 × 27,620 px	Extensive object categories (18). Oriented bounding boxes (OBB) for precise annotation. Includes instance difficulty scores. Widely adopted in international challenges (e.g., LUAI).	Extreme image size variation complicates processing. Complex and costly annotation process. High computational demands for full dataset utilization.
xView [251]	1M+ instances, 1400 km^2^	0.3 m/pixel	Exceptionally high resolution (WorldView-3). Unprecedented class diversity (60 object types). Supports few-shot learning and domain adaptation research. Designed for humanitarian and disaster response applications.	Pronounced class imbalance (“long-tail” distribution). Significant computational load due to data volume and resolution. Highly complex and expensive three-stage annotation pipeline.
VEDAI [254]	3700+ vehicles	Multiple Resolutions	Specialized focus on small vehicle detection. Unique multi-modal data (color and infrared spectra). Diverse conditions: occlusion, orientation, and lighting variations. Designed for challenging, unconstrained environments.	Limited scale compared to contemporary benchmarks. Extremely challenging; baseline performance remains low. Restricted to the vehicle category only.
DIOR [256]	192,472 instances, 23,463 images	Varied Resolutions	Comprehensive object coverage (20 categories). Exceptional diversity in weather, season, and resolution. Significant intra-class variation and inter-class similarity. Highly effective for robust evaluation of data-driven methods.	Geographic diversity may not fully represent global variation. Potential under-representation in some object classes. Limited compared to newer datasets in total scale.
INRIA [258]	810 km^2^ coverage	0.3 m/pixel	Rigorous generalization testing (train/test on different cities). Focus on building/not-building segmentation. High-resolution orthorectified imagery. Covers diverse urban settlements across Europe and North America.	Limited to a binary classification task. Test set labels not publicly available (online evaluation only). Focuses primarily on urban environments.

**Table 12:** Comparative analysis of key face detection datasets.

Dataset	Key Contributions and Importance	Advantages	Limitations
WIDER Face [262]	Bridges the critical gap between laboratory performance and real-world requirements. Set a new standard for scale and difficulty, becoming an essential benchmark for evaluating robust, real-world systems.	Massive scale; an order of magnitude larger than predecessors. Rich annotations for pose, occlusion, and lighting. Covers numerous real-world event categories.	Potential biases in event-based categorization. Lack of identity labels limits utility for recognition tasks. Extreme cases (small faces, heavy occlusion) remain highly challenging.
Labeled Faces in the Wild (LFW) [267]	A landmark benchmark that pioneered the study of unconstrained face verification. Provides standardized splits for direct method comparison, significantly accelerating research progress.	Carefully curated for real-world variability (pose, lighting, expression). Streamlined evaluation protocol with matched/mismatched pairs.	Demographic bias (celebrity-focused, Western origin). Relatively small scale for modern deep learning. Not suitable for studying isolated variables.
MAFA (Masked Faces) [263]	Pioneered large-scale research on occluded face detection. Provides detailed annotations for precise occlusion analysis. Critical for applications in health and surveillance.	Large-scale and focused on a critical challenge. Comprehensive attribute annotations (orientation, severity, type). High-quality labels ensured via cross-validation.	Specialized focus on occlusion limits its general-purpose use. Occlusion types may not cover all real-world scenarios.

**Table 15:** Cross-method synthesis of challenges, existing solutions, and remaining limitations across detector families.

Challenge	Single-Stage	Two-Stage	Transformer	Open-Vocab/LLM	Remaining Limitation
Long-tail data	Moderate	High	High	Low (zero-shot)	Rare classes still underperform; zero-shot gains uneven across domains
Dataset bias	High	High	Moderate	Low	Demographic and geographic gaps persist in benchmarks
Annotation cost	High	High	Moderate	Low (prompt-based)	Label noise and coverage gaps remain in specialized domains
Computational cost	Low	High	Very high	Very high	Accuracy–efficiency trade-off unresolved on edge devices
Small object detection	Moderate	High	Moderate–High	Moderate	Dense and tiny objects remain difficult under occlusion
Real-time deployment	Excellent	Poor	Moderate	Moderate	LLM latency still prohibitive for embedded systems
Reliability/calibration	Moderate	Moderate	Moderate	Low	Calibration under domain shift largely unverified

## Data Availability

The data presented in this study are available in the cited articles and publicly available datasets referenced throughout the manuscript. No new data were generated or analyzed in this study.
